# Nanozymes Integrated Biochips Toward Smart Detection System

**DOI:** 10.1002/advs.202519136

**Published:** 2025-12-19

**Authors:** Dongyu Chen, Wang Zheng, Zhihui Zhang, Shenping Yu, Xinxin Hang, Han Wu, Xiao‐Wei Xiang, Wei Mu, Yanli Jiao, Zaizai Dong, Lingqian Chang

**Affiliations:** ^1^ School of Engineering Medicine Beihang University Beijing 100191 China; ^2^ School of Biological Science and Medical Engineering Beihang University Beijing 100191 China; ^3^ Qingdao Research Institute Beihang University Qingdao 266100 China; ^4^ Department of Biomedical Engineering City University of Hong Kong Kowloon Hong Kong 999077 China

**Keywords:** artificial intelligence, biochips, multimodal biosensing, nanozymes, smart detection

## Abstract

Nanozyme integrated‐biochip systems merge the robust catalytic properties of nanozymes with the portability capabilities of biochips, which have demonstrated significant potential for molecular identification and diagnostic applications. Benefiting from the progressive incorporation of artificial intelligence (AI), nanozyme‐biochip systems further achieve substantial improvements in both efficiency and accuracy. In this review, recent progress in nanozyme‐biochip systems for intelligent detection are summarized. Advancing from fundamental concepts to integrated systems, this overview examines nanozyme‐driven signal amplification, biochip‐mediated signal presentation, and AI‐accelerated signal processing in nanozyme‐biochip platforms. Furthermore, the translational potential of nanozyme‐biochip systems is illustrated through a critical evaluation of their representative applications in clinical diagnostics, food safety, and environmental monitoring. The current major challenges and future directions in nanozyme‐biochip systems are also analyzed, with particular emphasis on AI‐assisted development. By integrating advances in nano‐catalysis, microdevice engineering, and intelligent computation, this review aims to provide an interdisciplinary roadmap for next‐generation biosensing systems.

## Graphical abstract

This review systematically outlines the integration of nanozymes, biochips, and artificial intelligence (AI) for intelligent biosensing. It details how their convergence enhances signal amplification, enables portable detection, and improves data interpretation. The work evaluates applications in clinical diagnostics, food safety, and environmental monitoring, discussing challenges and future directions for developing adaptive, multimodal platforms.

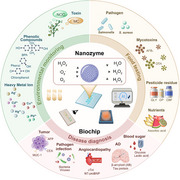

## 1 Introduction

Nanozymes are catalytic nanomaterials that mimic the functions of natural enzymes. Their combination of high stability and low cost presents a promising strategy to overcome the limitations of natural enzyme‐based biosensing technologies.^[^ ^1^, ^2^ ^]^ To harness the catalytic properties, nanozymes are increasingly integrated into biochip platforms, forming the “nanozyme‐biochip system”. In these systems, nanozymes evolve from individual catalytic units into system‐level components, which not only facilitate signal amplification but also leverage the advantages of biochips, including high‐throughput, portability, and automation.^[^ ^3^, ^4^, ^5^, ^6^ ^]^ This integration addresses other drawbacks of conventional methods, including the reliance on bulky laboratory equipment and complex operation procedures.^[^ ^7^, ^8^ ^]^ Building upon this foundation, the integration of artificial intelligence (AI) further empowers nanozyme‐biochip systems with capabilities such as intelligent signal interpretation, which effectively overcomes the inefficient manual data processing of traditional approaches and paves the way for real‐time, multi‐parameter analysis in complex conditions.^[^ ^9^, ^10^, ^11^ ^]^

**Figure 1:**
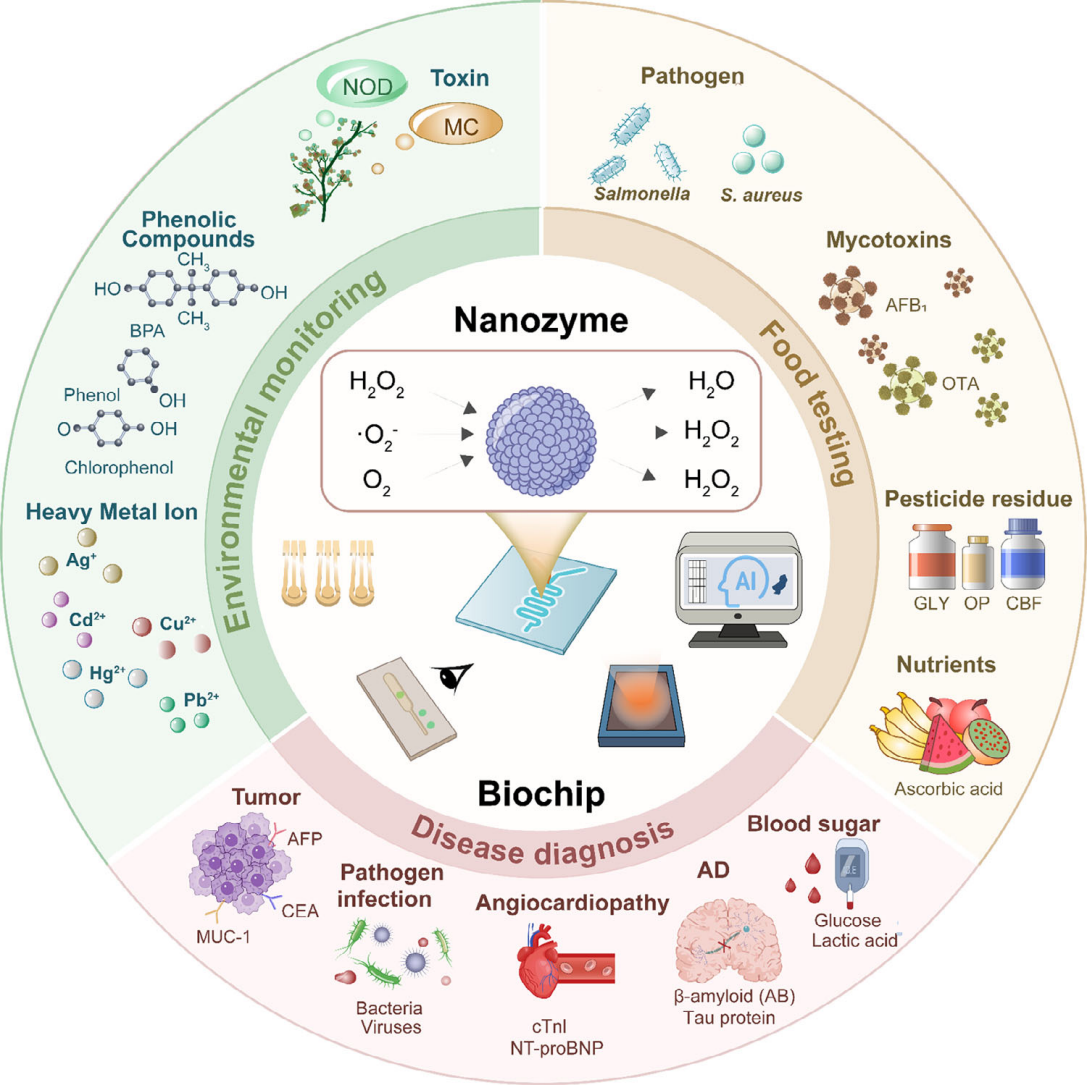
Schematic overview of nanozyme‐biochip systems and their representative applications across disease diagnosis, food testing and environmental monitoring.

Advances in recent nanozyme‐biochip systems have demonstrated high adaptability and scalability across a wide array of application scenarios (**Figure** **1**).^[^ ^12^ ^]^ Specifically, in clinical diagnostics, nanozyme‐biochip systems enable sensitive electrochemical or fluorescent detection of biomarkers associated with diabetes, cardiovascular and cerebrovascular diseases, infections, and tumors.^[^ ^13^, ^14^ ^]^ In this process, AI plays a vital role in baseline correction, response classification, and personalized health decision‐making. In food safety analysis, nanozyme‐biochip systems amplify weak fluorescence signals from bacterial contaminants, mycotoxins, or pesticide residues, and AI algorithms contribute to multichannel data interpretation and anomaly detection, ensuring both sensitivity and reliability in complex food matrices.^[^ ^15^ ^]^ In environmental monitoring, nanozyme‐biochip systems catalyze chromogenic reactions that generate colorimetric signals to detect heavy metal ions, phenolic pollutants, and algal toxins. Meanwhile, AI‐assisted image processing enables accurate quantification and real‐time risk assessment, even under field conditions.^[^ ^16^ ^]^ Together, these scenario‐specific applications underscore the unique capability of nanozyme‐biochip systems integrated with AI for intelligent biosensing.

In this review, we present a systematic and forward‐looking perspective on the convergence of nanozyme catalysis, biochip engineering, and AI in the context of smart biosensing. It highlights how the convergence of these components enables signal amplification, real‐time processing, and adaptive decision‐making across diverse detection scenarios. Representative application fields, including clinical diagnostics, food safety and environmental monitoring, are systematically categorized and critically analyzed. Furthermore, current limitations in transition from laboratory to commercial deployment are discussed, and future directions are proposed for the development of next‐generation biosensing platforms featuring ultra‐high sensitivity and in situ quantitative capability. By capturing the synergistic advantages of catalysis, microdevice integration, and computational intelligence, this review provides insights into next‐generation detection systems.

## 2 Nanozymes

Nanozymes are nanomaterials that mimic the catalytic activity of natural enzymes. Since Gao et al. first reported the peroxidase (POD)‐like activity of Fe_3_O_4_ nanoparticles in 2007, nanozymes have rapidly developed into a major subdiscipline of artificial enzyme research.^[^ ^17^ ^]^ Compared to natural enzymes, nanozymes offer advantages including facile synthesis, tunable structure/function, low production cost, and high environmental stability, emerging as powerful catalysts for developing highly sensitive and stable biosensing platforms.^[^ ^18^, ^19^ ^]^ In this chapter, we systematically review advances in nanozyme development, categorized according to their distinct catalytic mechanisms. Furthermore, several strategies for improving catalytic performance were evaluated from the perspective of their underlying catalytic mechanisms.

### 2.1 Oxidoreductase‐Like Nanozymes

Oxidoreductase‐like nanozymes catalyze electron‐transfer reactions through mechanisms resembling natural redox enzymes.^[^ ^20^ ^]^ These systems include: (i) POD‐like and catalase (CAT)‐like nanozymes for hydrogen peroxide (H_2_O_2_) decomposition; (ii) Glutathione peroxidase (GPx)‐like nanozymes for glutathione (GSH)‐mediated peroxide reduction; (iii) haloperoxidase (HPO)‐like nanozymes for halide oxidation; (iv) superoxide dismutase (SOD)‐like nanozymes for superoxide dismutation; and (v) oxidase (OXD)‐like nanozymes for oxygen‐coupled substrate oxidation. Their catalytic centers, typically featuring transition metal complexes or engineered nanomaterials, stabilize reactive intermediates during electron transfer processes. These redox mimics are governed by electron mobility, redox potential alignment, and radical stability‐key factors determining their catalytic performance.^[^ ^21^ ^]^ The following sections detail six major categories of oxidoreductase‐mimicking nanozymes, focusing on their unique electron‐transfer mechanisms and representative materials.

#### 2.1.1 Peroxidase (POD)‐like nanozymes

**Figure 2:**
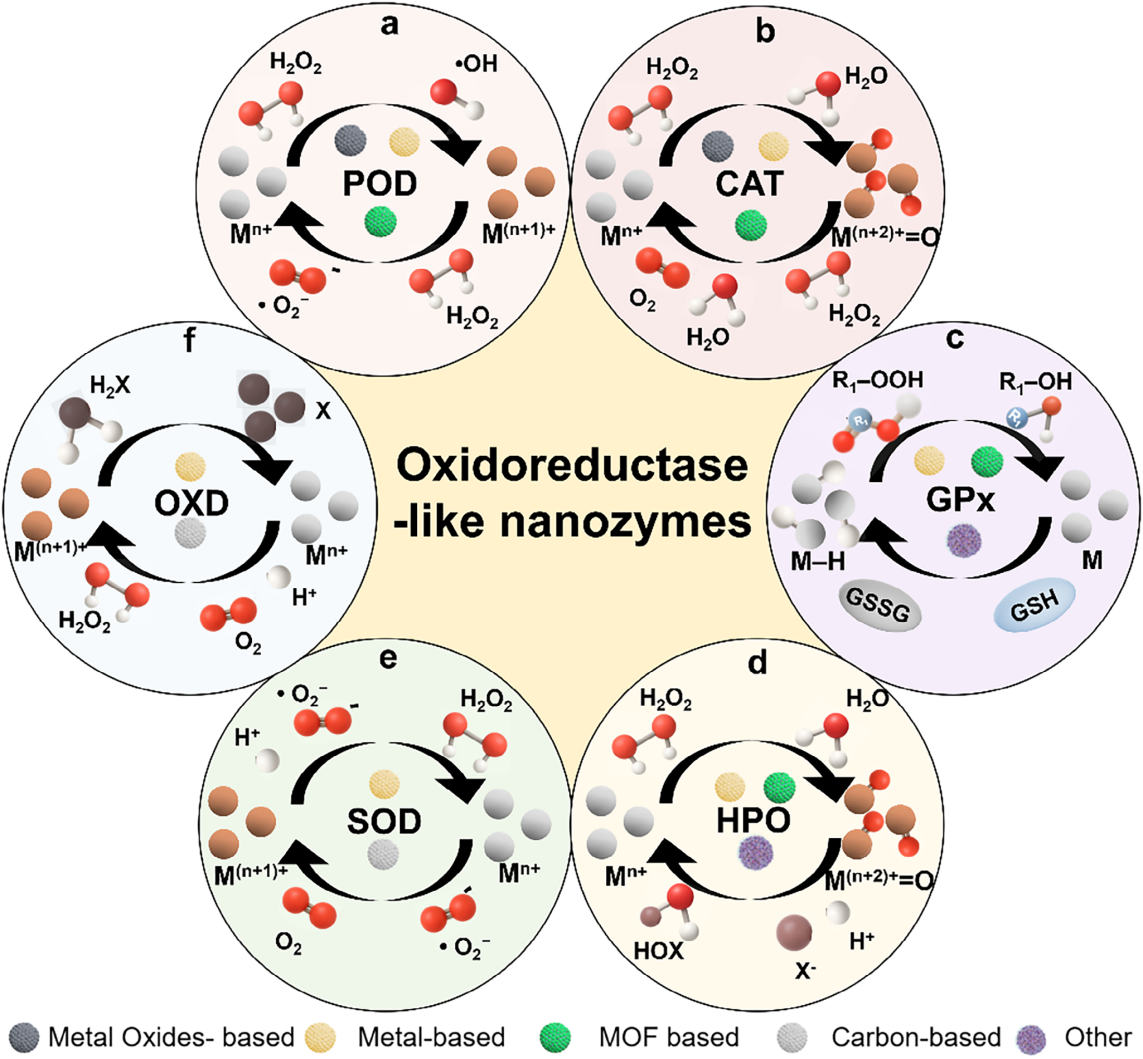
The schematic diagram of catalytic mechanism for oxidoreductase‐like nanozymes. a) POD‐like nanozymes catalyze H**_2_** O**_2_** via Fenton‐like reactions to generate ·OH, which oxidizes the substrate. b) CAT‐like nanozymes catalytically decomposes H**_2_** O**_2_** into H**_2_** O and O**_2_**, eliminating H**_2_** O**_2_**. c) GPx‐like nanozymes catalyzes GSH to GSSG and decomposes ROOH into harmless alcohols. d) HPO‐like nanozymes catalyzes the conversion of H**_2_** O**_2_** into HOX in the presence of halide ions (X**^−^**). e) SOD‐like nanozymes catalyzed **•**O**_2_^−^** undergoes a disproportionation reaction to produce H**_2_** O**_2_** and O**_2_**. f) OXD‐like nanozymes catalytically oxidizes substrates with O**_2_**, often accompanied by the formation of H**_2_** O**_2_** or H**_2_** O.

POD‐like nanozymes decompose H_2_O_2_ through a Fenton‐type electron transfer cycle centered on transition metal active sites (e.g., Fe, Ce, Mn) (**Figure** **2**a).^[^ ^22^, ^23^, ^24^ ^]^ The process begins with H_2_O_2_ adsorption onto a reduced metal ion (M^n+^), followed by a single electron transfer that cleaves the O–O bond. This reaction generates a highly reactive hydroxyl radical (•OH) and a hydroxide ion (OH^−^), while oxidizing the metal to a higher state (M^(n+1)+^). The •OH serves as the primary oxidant in peroxidase‐mimicking applications such as biosensing. To sustain catalytic activity, the nanozyme undergoes regeneration via a second electron transfer, where another H_2_O_2_ molecule acts as a reductant. This step returns the metal to its original M^n+^ state while producing a superoxide radical (O_2_•^−^) and protons. By cycling between oxidation states, the nanozyme continuously decomposes H_2_O_2_ and supplies •OH, enabling efficient oxidative reactions. This redox loop effectively mimics the function of natural peroxidases, leveraging robust electron‐transfer pathways to drive catalytic applications.^[^ ^25^, ^26^ ^]^

POD‐like nanozymes are built from a broad set of nanomaterials, including transition‐metal oxides (Fe/Co/Mn/Cu), noble‐metal nanoparticles (Au, Pt), conductive carbons, metal–organic frameworks, and Fe–N–C single‐atom catalysts.^[^ ^27^, ^28^, ^29^, ^30^, ^31^, ^32^, ^33^ ^]^ For transition‐metal oxides, Pt‐decorated Fe_3_O_4_ shows lower K_m_ (0.147 mM vs 0.485 mM for Fe_3_O_4_) and higher kcat (84.1 s^−1^ *vs* 66.6 s^−1^) in 3, 3', 5, 5'‐Tetramethylbenzidine (TMB) /H_2_O_2_ assays, yielding a ≈4.2× increase in k_cat_/K_m_ (5.72×10^5^ vs 1.37×10^5^ M^−1^·s^−1^).^[^ ^34^ ^]^ Among noble‐metal nanozymes, SiO_2_@Au maintained essentially unchanged POD‐like activity over 30 days at 25 °C.^[^ ^35^ ^]^ For conductive carbons, nitrogen‐doped graphene quantum dots (N‐GQDs) provide quantitative sensing figures which shows a linear range of 20–1170 µM with a limit of detection (LOD) of 5.3 µM for colorimetric H_2_O_2_ detection.^[^ ^36^ ^]^ MOFs exhibit high apparent activity, which relies on efficient substrate enrichment and electron transfer within their porous structure. For instance, a nano‐sized Cu‐MOF catalyzes standard TMB/H_2_O_2_ and o‐Phenylenediamine (OPDA) /H_2_O_2_ reactions, enabling the quantification of H_2_O_2_ (5–300 µM) and glucose (50–500 µM) with reliable linearity.^[^ ^37^ ^]^ In addition, single‐atom Fe–N–C catalysts can achieve specific activities that approach enzyme‐like levels. For instance, one reported Fe–N–C single‐atom nanozymes (SANs) exhibits a specific activity of 57.76 U/mg, which is comparable to horseradish peroxidase (HRP), while also demonstrating superior storage stability and robustness under harsh conditions.^[^ ^32^ ^]^ This high performance stems from their atomically dispersed Fe–N_x_ active sites on MOF‐derived porous carbon. Together, these materials set new standards for nanozyme performance and enable highly sensitive TMB or 2, 2′‐azino‐bis(3‐ethylbenzothiazoline‐6‐sulfonic acid) (ABTS) colorimetric detection.

#### 2.1.2 Catalase (CAT)‐Like Nanozymes

CAT‐like nanozymes mimic the fundamental function of natural catalase by efficiently catalyzing the decomposition of H_2_O_2_ into water and molecular oxygen (**Figure** 2b).^[^ ^38^ ^]^ The catalytic process begins with H_2_O_2_ binding at the enzyme‐mimicking active site, generally composed of redox‐active metal centers (Fe, Mn, Ce) or engineered nanoclusters. This binding facilitates the heterolytic cleavage of the O‐O bond, generating a metal‐hydroxide intermediate. Subsequently, a second H_2_O_2_ molecule interacts with this intermediate through an oxidation‐reduction pathway, producing oxygen gas and regenerating the catalytic site. This cyclic mechanism enables sustained H_2_O_2_ decomposition, making CAT‐like nanozymes particularly valuable for mitigating oxidative stress in biological systems.

CAT‐like nanozymes are constructed from cerium oxide nanoparticles, iron‐based metal–organic frameworks, and platinum alloy nanocrystals. For cerium oxide nanoparticles, the catalase‐like activity scales with the surface Ce^3^⁺ fraction and H_2_O_2_ adsorption, showing Langmuir‐type behavior and a unified scaling relationship.^[^ ^39^ ^]^ Iron‐based MOFs achieve remarkable oxygen production rates of 3.8 mmol g^−1^ h^−1^ while operating effectively across broad physiological pH ranges.^[^ ^40^ ^]^ They offer a unique platform by providing framework‐confined redox sites and an in situ oxygen supply. For instance, the porphyrinic Fe centers in Hf‐DBP‐Fe generate oxygen (O_2_) from H_2_O_2_ at a turnover frequency of ≈8.7 h^−1^. Moreover, multiple Fe‐MOFs have demonstrated intrinsic CAT‐like dismutation activity in aqueous conditions relevant for biosensing and therapeutic applications.^[^ ^41^ ^]^ Platinum nanozymes simultaneously catalyze H_2_O_2_ dismutation and scavenge O_2_•^−^/ singlet oxygen (^1^O_2_), while demonstrating sustained activity across an exceptionally wide pH range (1.1–11) and at physiologically relevant temperatures.^[^ ^42^ ^]^ These nanomaterial systems enable robust signal transduction in electrochemical or optical sensing platforms by leveraging their efficient catalytic decomposition of peroxides.

#### 2.1.3 Glutathione Peroxidase (GPx)‐Like Nanozymes

GPx‐like nanozymes operate through a distinct catalytic mechanism that mimics the native enzyme's ability to reduce peroxides using glutathione (GSH).^[^ ^43^ ^]^ Unlike peroxidase‐mimics that generate reactive radicals, GPx‐nanozymes function as redox regulators by catalyzing the conversion of peroxides (H_2_O_2_, ROOH) into products (H_2_O, ROH) while oxidizing GSH to oxidized glutathione (GSSG). The catalytic process relies on specific elements (Se, Te, V) or defect sites that undergo reversible redox transitions (**Figure** 2c).^[^ ^44^ ^]^ The reaction initiates with peroxide activation on the nanozyme's active site, leading to the oxidation of its catalytic center (e.g., Se or low‐valence metal species) and the simultaneous reduction of the peroxide. The oxidized nanozyme then interacts with GSH, forming a transitional enzyme‐glutathione complex. This complex is subsequently reduced by a second GSH molecule, regenerating the active site and releasing GSSG.

Typical GPx‐like nanozymes encompass several material classes: selenium‐ or tellurium‐containing nanostructures, vanadium‐based oxides, and sulfur‐functionalized metal–organic frameworks.^[^ ^45^ ^]^ Selenium‐containing nanostructures, including Se‐functionalized carbon dots and Se‐enriched carbon quantum dots, display GPx‐like activity in standard glutathione assays and afford cytoprotection in oxidative‐stress models.^[^ ^46^ ^]^ In parallel, tellurium‐containing materials have recently been translated into nanoarchitectures that exhibit thiol‐peroxidase/GPx‐like catalysis in water, underscoring the broader chalcogen‐centered design space for GPx nanozymes.^[^ ^47^ ^]^ Beyond chalcogens, vanadium‐based oxides provide substrate‐versatile GPx‐like catalysis toward both H_2_O_2_ and lipid hydroperoxides, with cellular antioxidant effects validated in multiple studies.^[^ ^45^ ^]^ Framework‐confined or ligand‐engineered MOF platforms (e.g., porphyrinic Fe‐MOFs, V‐MOFs) offer tunable redox microenvironments and GSH‐accessible channels to support GPx‐like reaction cycles.^[^ ^44^ ^]^ These diverse GPx‐like nanozymes serve as powerful catalysts for colorimetric, fluorescent, and electrochemical biosensing, enabling the detection of various peroxides and thiols by transducing their catalytic reactions into quantifiable signals.

#### 2.1.4 Haloperoxidases (HPO)‐Like Nanozymes

HPO‐like nanozymes mimic the functionality of natural haloperoxidases in catalyzing the oxidation of halide ions (Cl^−^, Br^−^, I^−^) in the presence of H_2_O_2_, leading to the formation of hypohalous acids (HOX) or related halogenating species (**Figure** 2d).^[^ ^48^ ^]^ The catalytic mechanism initiates with the activation of H_2_O_2_ at the nanozyme's active site, typically comprising transition metal centers (V, Mo, Mn) or specially designed single‐atom configurations. This activation involves heterolytic cleavage of the peroxide O‐O bond, forming a metal‐oxo intermediate. Subsequently, the activated oxygen species reacts with halide ions through a two‐electron oxidation process, generating hypohalous acid. The catalytic cycle completes with the regeneration of the original active site, enabling continuous halogenation activity.^[^ ^21^, ^49^ ^]^

HPO‐like nanozymes have been developed using vanadium oxide nanostructures, molybdenum disulfide materials, cerium‐based compounds, and polyoxometalates. Among representative systems, V_2_O_5_ nanoarchitectures enable in situ generation of HOBr for biological modeling applications, while peroxovanadium complexes demonstrate third‐order reaction kinetics at pH 6.5 with a rate constant k of 2.49 × 10^5^ dm^6^·mol^−2^·s^−1^.^[^ ^50^ ^]^ Beyond vanadates, transition‐metal engineering of defect‐rich chalcogenides (e.g., MoS_2_) enables efficient haloperoxidase‐mimicking activity, broadening the spectrum of HPO‐like nanozymes from conventional oxides to versatile chalcogenide platforms.^[^ ^51^ ^]^ Leveraging oxygen vacancy and lattice redox mechanisms, cerium‐based materials including **CeO_2‐x_** nanorods and Ce‐MOFs demonstrate stable recyclability in repeated catalytic cycles for the aqueous‐phase phenol red/Br^−^/H_2_O_2_ bromination reaction.^[^ ^52^, ^53^ ^]^ In addition, polyoxometalates (POMs), including W/Mo/V‐containing clusters, offer acid‐stable halide‐oxidation platforms and can be defect‐modulated (e.g., oxygen‐vacancy‐rich POMs) to sustain HPO‐like catalysis under acidic pH.^[^ ^54^ ^]^ These material families establish robust halogenation under mild aqueous conditions, providing versatile routes to on‐demand HOX generation for sensing.^[^ ^55^ ^]^

#### 2.1.5 Superoxide Dismutase (SOD)‐Like Nanozymes

SOD‐like nanozymes mimic the function of superoxide dismutase by catalyzing the dismutation of superoxide anion (•O_2_^−^) into H_2_O_2_ and O_2_ (**Figure** 2e).^[^ ^17^ ^]^ Representative SOD‐like nanozymes are predominantly metal oxides.^[^ ^56^ ^]^ Among these, CeO_2_ is a prototypical SOD mimic, with mixed Ce^3+^/Ce^4+^ states and oxygen vacancies enabling rapid redox cycling.^[^ ^57^ ^]^ Taking CeO_2_ nanozymes as a representative example, their catalytic mechanism initiates with the adsorption of superoxide anions onto oxygen vacancy sites associated with Ce^3+^ cations.^[^ ^58^ ^]^ During the reaction, Ce^3+^ sites donate electrons to reduce •O_2_^−^ to O_2_, while neighboring Ce^4+^ sites oxidize another •O_2_^−^ into O_2_^2−^, which subsequently combines with protons to form H_2_O_2_. The rapid Ce^3+^/Ce^4+^ interconversion regenerates active centers continuously, closely mimicking the natural Mn/Zn‐SOD pathway. Surface oxygen vacancies facilitate electron transfer and lower the activation barrier for •O_2_^−^ adsorption and dismutation.^[^ ^59^, ^60^ ^]^

Beyond these metal oxides, SOD‐like nanozymes have been expanded to encompass a diverse range of nanomaterials. Based on the composition and structural characteristics, they can be categorized into four classes: carbon‐based materials, noble metal‐based materials, metal compounds, and metal‐organic frameworks. Carbon‐based materials, including heteroatom‐doped graphene quantum dots, serve as effective scavengers of •O_2_^−^ in both cellular and biochemical models. Noble‐metal‐based materials such as Pt nanoparticles effectively quench •O_2_^−^ and decompose H_2_O_2_ in a dose‐dependent fashion. Metal compounds demonstrate robust multi‐enzyme antioxidant capacity, with MnO_2_@GO achieving 95.5% inhibition under optimized conditions. MOF‐based systems (e.g., CuZn‐ZIF‐8) exhibit efficient SOD‐like activity by utilizing framework‐confined Cu/Zn dual sites that mimic the structural and functional features of natural CuZn‐SOD enzymes.^[^ ^61^, ^62^, ^63^, ^64^ ^]^ These nanozymes enable sensitive reactive oxygen species (ROS) detection by leveraging their superoxide scavenging and multi‐enzyme antioxidant activities for biosensing applications.

#### 2.1.6 Oxidase‐Like (OXD) Nanozymes

OXD‐like nanozymes mimic natural oxidases by catalyzing the dehydrogenation of amino substrates and in situ reduction of O_2_ to H_2_O_2_, and in some cases directly to H_2_O, thereby enabling self‐sufficient cascade reactions without exogenous H_2_O_2_ (Figure 2f).^[^ ^65^ ^]^ The catalytic mechanism typically initiates with the activation of molecular oxygen at the nanomaterial's active site, which commonly consists of transition metal coordination centers (Cu, Co, Fe) or nitrogen‐doped carbon structures.^[^ ^66^ ^]^ This activation process facilitates the reduction of oxygen to either hydrogen peroxide via a two‐electron transfer pathway or directly to water through a four‐electron route, depending on the specific nanozyme architecture. Concurrently, substrate molecules undergo oxidation through electron abstraction, with the generated electrons being shuttled to the activated oxygen species. The continuous regeneration of active sites during this redox cycle enables sustained oxidase‐mimetic activity.^[^ ^20^ ^]^

Representative systems span copper/iron single‐atom assemblies, cobalt nitrogen carbon hybrids, noble‐metal hollow or porous structures, and Fe–N–C graphene architectures. A quantitative benchmark is the porous Fe–N_3_ single‐atom nanozyme (pFeSAN), which exhibits 3.3 fold and 8791 fold higher OXD‐like activity than Fe–N_4_ and Fe_3_O_4_ nanozymes, respectively. This superior performance is attributed to its maximized active‐site exposure and highly efficient O_2_ activation at the Fe–N_3_ moieties.^[^ ^67^ ^]^ Within Co–N–C platforms, Co on N‐doped carbon steer molecular oxygen through the 2e^−^ reduct ion pathway to H_2_O_2_, enabling applications in colorimetric detection systems such as TMB‐based analyte assays.^[^ ^68^ ^]^ Noble‐metal nanozymes (e.g., Pt) are capable of oxidizing chromogenic substrates in the absence of added H_2_O_2_ while exhibiting robust and recyclable performance in aqueous environments.^[^ ^69^ ^]^ In Fe–N–C graphene frameworks, the active sites exhibit high stability during repeated measurement cycles, facilitating their use in inhibition‐based detection systems.^[^ ^70^ ^]^ Together, these materials provide adaptable OXD catalysis in bioanalysis and oxidative biocatalysis.

### 2.2 Hydrolase‐Like Nanozymes

Hydrolase‐like nanozymes mediate the cleavage of covalent bonds through acid‐base catalysis or nucleophilic substitution, following mechanisms analogous to those of natural hydrolases.^[^ ^71^ ^]^ Typical hydrolytic pathways involve i) phosphatase‐like nanozymes for phosphate ester hydrolysis; ii) nuclease‐like systems for phosphodiester bond cleavage; iii) glycosidase‐like mimics for glycosidic bond scission; iv) lipase‐like catalysts for ester bond hydrolysis; and v) protease‐like analogues for peptide bond cleavage.^[^ ^71^ ^]^ These reactions proceed via proton transfer or nucleophilic attack on electrophilic centers, forming transient tetrahedral intermediates stabilized by metal‐oxide Lewis acid sites or coordinated hydroxyl groups.^[^ ^72^ ^]^ Compared with redox‐type nanozymes, hydrolytic mimics rely less on electron transfer and more on surface polarity, hydration energy, and coordination geometry of active sites, which govern their catalytic specificity and efficiency.^[^ ^73^ ^]^ In the following sections, we classify hydrolase‐like nanozymes into five main groups to introduce their catalytic mechanisms and representative materials.

#### 2.2.1 Phosphatase‐Like Nanozymes

**Figure 3:**
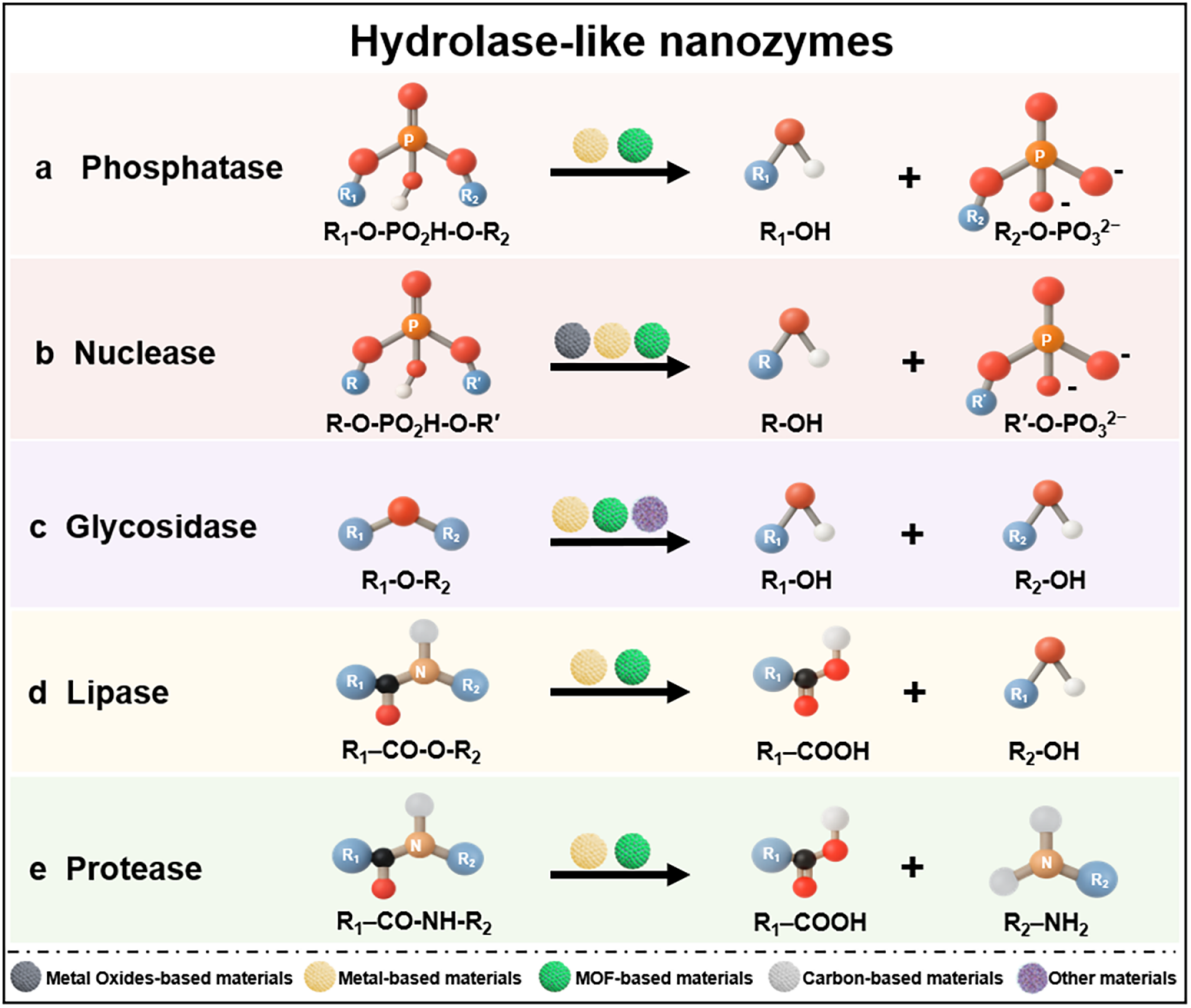
Hydrolase‐like nanozymes, which catalyze hydrolytic cleavage of phosphate esters, nucleic acids, glycosidic linkages, or peptide bonds. a) Phosphatase‐like nanozymes hydrolyze phosphate esters (R_1_–O–PO_2_H–O–R_2_), generating alcohols (R_1_–OH) and phosphate‐containing products (R_2_–O–PO_3_^2−^). b) Nuclease‐like nanozymes cleave phosphodiester linkages in nucleic acids (R–O–PO_2_H–O–R′), producing nucleoside alcohols (R–OH) and phosphate‐terminated fragments (R′–O–PO_3_^2−^). c) Glycosidase‐like nanozymes hydrolyze glycosidic bonds (R_1_–O–R_2_), resulting in the formation of two monosaccharide‐derived alcohols (R_1_–OH and R_2_–OH). d) Lipase‐like nanozymes hydrolyze ester bonds (R_1_–CO–O–R_2_), yielding fatty‐acid products (R_1_–COOH) and alcohols (R_2_–OH). e) Protease‐like nanozymes hydrolyze peptide bonds (R_1_–CO–NH–R_2_), producing carboxyl‐terminated fragments (R_1_–COOH) and amino‐containing fragments (R_2_–NH_2_).

Phosphatase‐like nanozymes, which replicate the hydrolytic cleavage of phosphoester bonds by natural alkaline and acid phosphatases, possess superior operational stability, high interference resistance, and tunable catalytic activity (**Figure** **3**a).^[^ ^74^ ^]^ The catalytic cycle usually starts with coordination and activation of the phosphoryl group at Lewis‐acidic metal centers (e.g., Zr^4+^, Ce^4+^), which polarize the P–O bonds and lower the barrier for nucleophilic attack. Nearby hydroxyl groups or bound water molecules then attack the phosphorus center to generate a metal‐stabilized tetrahedral transition state. Subsequent proton transfer and collapse of this intermediate promote departure of the leaving group and formation of the dephosphorylated product. Hydrolysis of the metal phosphate adduct regenerates the active Lewis‐acid site and restores the nucleophilic hydroxyl, thereby closing the catalytic cycle. Through this cooperative Lewis acid–base mechanism, phosphatase‐like nanozymes stabilize high‐energy transition states and accelerate phosphoester bond scission in a manner analogous to natural phosphatases.

Phosphatase‐like nanozymes have been designed using zirconium‐based metal–organic frameworks (MOFs), defect‐engineered metal oxides, and other inorganic nanostructures. This activity achieves reaction rates between 10^−3^ and 10^−4^ per second for model peptide substrates while exhibiting broad protease‐like hydrolysis capability.^[^ ^72^ ^]^ Beyond MOFs, metal oxide nanozymes offer another major family of phosphatase mimics. Defect‐rich CeO_2_ nanoarchitectures combine basic surface hydroxyls with Lewis‐acidic Ce centers, affording robust and recyclable p‐nitrophenyl phosphate (pNPP) dephosphorylation. Grain‐boundary‐rich “ceria metallenes” further enhance this structure–activity relationship by increasing the density of cooperative basic and acidic sites.^[^ ^75^ ^]^ Other inorganic nanomaterials can also be structurally engineered to achieve efficient phosphoester hydrolysis. For instance, the precise incorporation of copper sites into UiO‐type nodes leverages their pore confinement effects to stabilize reaction transition states, significantly enhancing the hydrolysis of both phosphate monoesters and diesters.^[^ ^76^ ^]^ This diverse family of materials collectively establishes a versatile and durable set of phosphatase‐like nanocatalysts, which also provides significant convenience for subsequent biosensing applications.

#### 2.2.2 Nuclease‐Like Nanozymes

Nuclease‐like nanozymes emulate natural nucleases by hydrolyzing phosphodiester bonds in DNA/RNA, exhibiting high efficiency, programmable specificity, and biostability (Figure 3b).^[^ ^77^, ^78^ ^]^ Their catalytic cycle usually begins with electrostatically driven substrate adsorption, during which the negatively charged nucleic acid backbone binds to positively charged surface sites. Lewis‐acidic metal centers (e.g., Ce^4+^, Zr^4+^) then activate coordinated water molecules to generate nucleophilic hydroxide ions. These activated nucleophiles attack the phosphorus atom within the phosphodiester linkage to form a pentacoordinate, trigonal bipyramidal transition state that is stabilized by metal coordination. Subsequent proton transfer to the leaving group, assisted by Brønsted‐acidic sites or coordinated water molecules, drives scission of the labile P–O bond and formation of shorter oligonucleotide fragments. Hydrolysis of the transient metal–phosphate adduct regenerates the Lewis‐acidic center and restores the nucleophilic water/hydroxide species, thereby closing the catalytic cycle. Through this cooperative acid–base mechanism, nuclease‐like nanozymes achieve controlled strand cleavage under mild aqueous conditions in a manner analogous to natural nucleases.

Nuclease‐like nanozymes have been engineered on diverse material platforms, including Zr(IV)‐cluster MOFs, CeO_2_‐based oxides, lanthanide catalysts, and photo‐addressable carbon materials. Zr_6_‐node MOFs, such as those from the MOF‐808, UiO, and NU series, hydrolyze model phospho(e)ster substrates in water. This reaction proceeds via node‐assisted activation of the phosphoryl group, achieving observed rate constants (*k*_obs_) on the order of 10^−2^–10^−1^ min^−1^.^[^ ^77^, ^78^ ^]^ Their catalytic performance can be further enhanced by heterometal doping strategies (e.g., Cu incorporation), which stabilize the transition state and increase turnover rates. As an oxide counterpart, CeO_2_ acts as a DNase I mimic by hydrolytically fragmenting single‐stranded DNA, a mechanism confirmed by mass spectrometry to exclude oxidative pathways. Its robust nuclease‐like activity in aqueous media stems from the Ce^3+^/oxygen‐vacancy density and polynucleotide adsorption at surface –OH groups.^[^ ^79^ ^]^ Beyond Zr/Ce systems, lanthanide catalysts furnish classical precedents for phosphodiester hydrolysis near physiological conditions (e.g., Ce^4+^ for DNA, Tm^3+^/Yb^3+^/Lu^3+^ for RNA), delineating general hard‐acid design rules now adopted in modern nanoarchitectures.^[^ ^80^ ^]^ Photo‐addressable carbon platforms (e.g., UV‐activated graphene oxide) demonstrate programmable strand scission via light‐triggered pathways that can be coupled to sequence‐recognition motifs to impart selectivity in space and time.^[^ ^81^ ^]^ Collectively, these diverse platforms significantly expand the functional toolbox for controlled nucleic acid cleavage, enabling advanced applications in biosensing.

#### 2.2.3 Glycosidase‐Like Nanozymes

Glycosidase‐like nanozymes facilitate the hydrolysis of glycosidic bonds (C–O) in sugar molecules through a concerted acid–base mechanism (Figure 3c). The catalytic cycle begins with protonation of the glycosidic oxygen by a Brønsted acid site, generating a carbocation‐like transition state. This intermediate is subsequently nucleophilically attacked by an activated water molecule or a surface µ‐OH group, followed by proton transfer to release the hydrolyzed product and regenerate the active site. In this way, glycosidase‐like nanozymes mirror the mechanism of natural glycosidases while operating under simple aqueous conditions.^[^ ^82^, ^83^ ^]^

Glycosidase‐like nanozymes have been developed on a range of material platforms such as Ce‐based MOFs, molecularly imprinted organic scaffolds, and Zn‐based single‐atom nanozymes. Ce‐FMA is a typical inorganic example that uses hard Lewis‐acid sites (Ce^4+^) together with nearby µ‐OH groups to stabilize the transition state.^[^ ^71^ ^]^ This cooperation enables efficient glycosidic bond hydrolysis and allows degradation of complex biological structures such as biofilms under mild conditions. As a complementary organic route, molecularly imprinted or self‐assembled synthetic glycosidases provide substrate‐ and linkage‐selective hydrolysis of oligo‐ and polysaccharides in water.^[^ ^84^ ^]^ Shape and charge complementarity within the imprinted pocket underpins this selectivity and supports catalyst recyclability. Zn‐based single‐atom nanozymes with planar ZnN_4_ motifs on N‐doped carbon have also been reported to promote glycosidic bond hydrolysis in aqueous media.^[^ ^85^ ^]^ These SANs offer an atomically well‐defined model for acid–base cooperativity on carbon supports and enable activity tuning through control of electronic structure and local polarity. These studies collectively define how glycanase‐like nanomolecules achieve highly efficient and tunable glycosidic bond hydrolysis through catalytic mechanisms. This facilitates the detection of relevant glycosylated biomarkers in biological assays, enabling subsequent signal amplification and sensitive detection.^[^ ^86^ ^]^

#### 2.2.4 Lipase‐Like Nanozymes

Lipase‐like nanozymes catalyze the hydrolytic cleavage of ester bonds through an interface‐activation mechanism (Figure 3d). Their activity is highest at boundaries between hydrophobic and hydrophilic phases, such as oil–water or solid–liquid interfaces. At these interfaces, the lipid substrate first anchors and orients its ester bond toward the catalytic site. A water molecule then acts as a nucleophile and attacks the ester carbonyl, generating a high‐energy tetrahedral transition state. Nearby hydrogen‐bond donors or Lewis‐acidic sites stabilize this anionic intermediate in a manner analogous to the “oxyanion hole” of natural lipases. Subsequent proton transfer and collapse of the intermediate release the hydrolysis products and regenerate the active site. Through this interfacial acid–base mechanism, lipase‐like nanozymes reproduce the key features of natural lipase catalysis under mild aqueous conditions.

Lipase‐like nanozymes have been realized on diverse material platforms, including amphiphilic self‐assembled architectures, minimalist peptide‐based catalysts, and engineered inorganic or metallic scaffolds. Amphiphilic self‐assembled structures such as peptide fibrils catalyze ester hydrolysis in water at room temperature.^[^ ^87^, ^88^ ^]^ In these systems, key residues such as histidine and the local hydrophobic microenvironment are crucial for stabilizing the transition state and positioning substrates at the interface. Minimalist peptide‐based catalysts, including short peptides and peptide–nanoparticle conjugates, further demonstrate that local hydrophobicity and nanoconfinement are sufficient to boost hydrolysis rates.^[^ ^89^ ^]^ These results show that complex protein scaffolds are not strictly required to achieve lipase‐like activity. Engineered inorganic scaffolds, such as mesoporous silica, can also create artificial hydrophobic pockets.^[^ ^90^, ^91^ ^]^ They operate in diverse media, including water–oil mixtures, and can be regenerated and reused. Similarly, metal nanoparticles with tailored surface ligands can catalyze ester hydrolysis via interfacial activation without relying on redox pathways. These diverse platforms demonstrate that tailored hydrophobicity, nanoconfinement, and interfacial activation are key to achieving efficient, recyclable lipase‐like catalysis in aqueous and multiphase environments.

#### 2.2.5 Protease‐Like Nanozymes

Protease‐like nanozymes catalyze the proteolytic cleavage of peptide bonds in proteins (Figure 3e). Their catalytic cycle typically starts when a metal center (e.g., Zr^4+^, Cu^2+^) coordinates to and polarizes the amide carbonyl (C = O) group. A water molecule or a µ‐OH group is then activated as a nucleophile and attacks the polarized carbonyl carbon. This step generates a high‐energy tetrahedral intermediate. Hydrogen bonding and electrostatic interactions within the confined nanoenvironment stabilize this intermediate. Subsequent collapse of the intermediate cleaves the peptide bond, releases the protein fragments, and regenerates the active metal site for the next catalytic cycle. In this way, protease‐like nanozymes mimic essential features of natural proteases while operating under mild aqueous conditions.

Zr‐cluster MOFs, Cu‐based crystalline MOFs, magnetic nanozymes, and metal–carbon hybrids have been explored as material platforms for protease‐like nanozymes. Zr‐cluster MOFs such as MOF‐808, NU‐1000, and MIP‐201 provide strongly Lewis‐acidic Zr^4+^ nodes that efficiently catalyze peptide bond cleavage in water.^[^ ^92^, ^93^ ^]^ Their pore size and local chemical environment are key determinants of catalytic activity and substrate selectivity, which makes these frameworks tunable nanoreactors. Crystalline Cu‐based MOFs such as HKUST‐1 offer open copper sites with intrinsic protease‐like activity and demonstrate that well‐defined inorganic lattices can hydrolyze proteins without biological enzymes.^[^ ^94^, ^95^ ^]^ Magnetic nanozymes such as CuFe_2_O_4_ also display protease‐like behavior, cleaving proteins such as bovine serum albumin (BSA) while enabling easy magnetic separation and reuse over multiple cycles. Metal–carbon hybrids, exemplified by Cu_2_O nanoparticles supported on carbon dots. Further show that combining metal centers with carbonaceous supports can create synergistic microenvironments that promote efficient peptide bond hydrolysis in aqueous solutions.^[^ ^96^ ^]^ These features collectively offer practical platforms for protein digestion and proteomics preprocessing without relying on fragile natural proteases.

### 2.3 Nanozymes with Other Enzymatic Activity

In addition to oxidoreductase‐ and hydrolase‐like nanozymes, researchers have recently extended the catalytic landscape of nanozymes toward lyase‐, isomerase‐, and ligase‐like systems. These emerging nanozymes imitate complex biochemical transformations that go beyond electron transfer or hydrolysis, such as non‐hydrolytic bond cleavage, intramolecular rearrangement, and energy‐assisted covalent coupling.^[^ ^97^, ^98^ ^]^ Although these systems are still in their infancy, they represent an important frontier in artificial enzyme research and demonstrate the expanding catalytic versatility of nanozymes.

#### 2.3.1 Lyase‐Like Nanozymes

**Figure 4:**
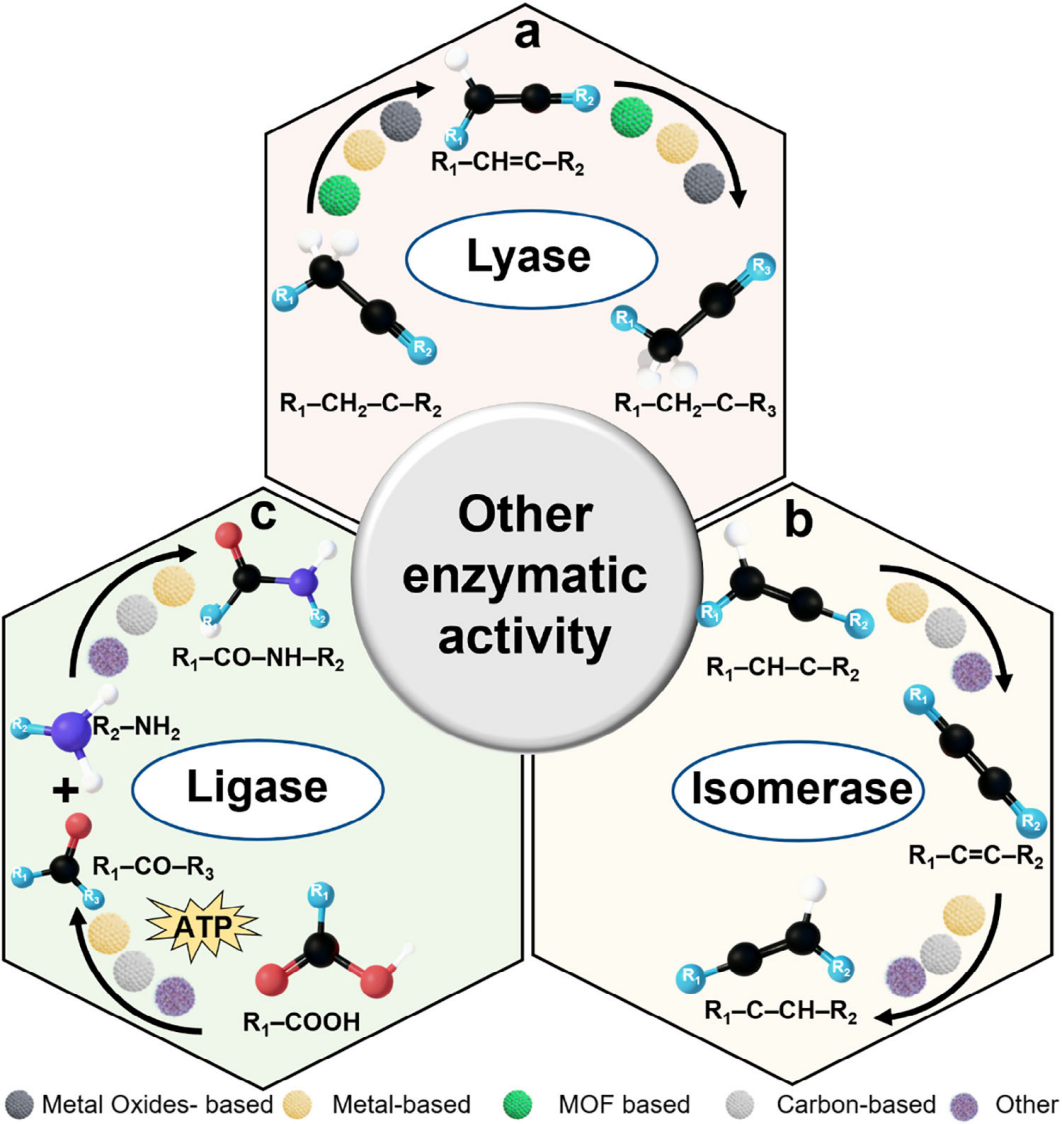
Non‐redox nanozymes, which catalyze the cleavage of chemical bonds in substrates, the rearrangement of their molecular structures, or the assembly of new compounds. a) Lyase‐like nanozymes catalyze non‐hydrolytic C–C bond cleavage or formation in substrates such as α‐hydroxy ketones (R_1_–CH_2_–C–R_2_) and enones (R_1_–CH = C–R_2_), converting saturated substrates into unsaturated products and vice versa. b) Isomerase‐like nanozymes mediate intramolecular rearrangements in alkyl‐substituted alkenes (R_1_–CH–CH–R_2_) and alkynes/enynes (R_1_–C = C–R_2_), producing structural isomers through internal bond reorganization. c) Ligase‐like nanozymes catalyze ATP‐driven amide‐bond formation between carboxylic acids (R_1_–COOH) and amines (R_2_–NH_2_), assembling new amide products such as peptide‐like structures (R_1_–CO–NH–R_2_).

Lyase‐like nanozymes catalyze the cleavage or formation of C–C, C–O, and C–N bonds through non‐hydrolytic pathways, such as decarboxylation reactions (Figure **4**a).^[^ ^98^ ^]^ They mimic natural lyases (EC 4) by combining basic or defect‐rich sites with metal–ligand coordination motifs on their surfaces. Basic sites deprotonate substrates and generate reactive intermediates, while metal centers stabilize anionic or radical species through coordination. Within nanoconfined environments, these elements work together to synchronize proton and electron transfer steps and to lower activation barriers. In this way, lyase‐like nanozymes reproduce key features of lyase‐class chemistry under mild aqueous or mixed‐solvent conditions.

Carbon‐based nano‐organocatalysts, self‐assembled peptide catalysts, and single‐atom Zn–N–C nanozymes have all been utilized as material bases for constructing lyase‐like nanozymes. Proline‐derived carbon nanoparticles act as nano‐organocatalysts that promote stereoselective aldol reactions in mixed water/DMF solvents.^[^ ^99^ ^]^ They achieve high conversions of ≈80–100% and excellent enantioselectivities of 96–98% ee, even after multiple reuse cycles. These results show that nanoscale carbon interfaces can support lyase‐like C–C bond formation with high stereocontrol. Self‐assembled peptide catalysts provide another soft nanoenvironment for lyase‐mimicking reactions.^[^ ^100^ ^]^ Peptide micelles or fibrils accelerate both aldol and retro‐aldol reactions through amine catalysis and stabilization of high‐energy transition states. Retro‐aldol rate enhancements can reach up to 10^4^‐fold relative to the uncatalyzed background, highlighting the impact of hydrophobic pockets and precisely positioned catalytic residues. In parallel, single‐atom Zn–N–C nanozymes that mimic carbonic anhydrase (EC 4.2.1.1) catalyze reversible CO_2_ hydration and dehydration under mild aqueous conditions with overall conversions above 91%.^[^ ^101^ ^]^ These materials demonstrate that minimal Zn–N_4_ motifs on carbon supports are sufficient to sustain lyase‐type chemistry on solid catalysts. These studies collectively demonstrate how tailored nanoscale microenvironments enable efficient, selective, and reusable lyase‐like catalysis to promote C–C bond formation and reversible gas hydration reactions.

#### 2.3.2 Isomerase‐Like Nanozymes

Isomerase‐like nanozymes catalyze reactions that rearrange atoms within a molecule or change its 3D shape without altering its chemical formula, effectively mimicking natural mutase and racemase enzymes (Figure 4b).^[^ ^102^ ^]^ Their catalytic power comes from specially designed active sites. These sites, crafted through techniques like molecular imprinting or metal‐organic frameworks, are chiral or asymmetric. When a substrate binds, these sites force it into a strained conformation and modify its electronic properties. This process is aided by weak interactions (like π‐π stacking) that stabilize the distorted transition state, while nearby acid or base groups help transfer protons or enable difficult bond rotations. These structural and electronic features constitute general design principles for constructing heterogeneous nanozyme architectures with isomerase‐like activity.

A variety of isomerase‐like nanozyme systems have emerged, ranging from Au nanoparticles that catalyze cis–trans isomerization of substituted azobenzenes to MOF‐based catalysts capable of glucose–fructose rearrangement.^[^ ^103^ ^]^ In parallel, MOFs (e.g., MIL‐101(Cr), UiO‐derived) further enable glucose fructose isomerization through Lewis‐acid/Lewis‐base cooperativity in confined pores under mild conditions, demonstrating solid‐state control of rearrangements.^[^ ^104^ ^]^ Collectively, these studies show that engineered chiral/asymmetric active sites plus nanoconfinement enable selective, enzyme‐like isomerization suited to stereoselective sensing and metabolite‐level reaction control.

#### 2.3.3 Ligase‐Like Nanozymes

Ligase‐like nanozymes catalyze covalent bond formation to join two molecular fragments, conceptually mirroring natural ligases used in nucleic‐acid and peptide assembly (Figure 4c). At nano‐interfaces, this is achieved by precisely aligning and bringing the reacting fragments close together, which reduces the entropic cost of the reaction. Metal‐ion coordination and hydrogen‐bond networks then help stabilize high‐energy intermediates during bond formation. In nucleic acid contexts, metal‐assisted activation of molecular termini can enable ligation chemistry without requiring the adenylation step typically used by protein‐based ligases.^[^ ^105^ ^]^

Ligase‐like nanozymes have been developed across nucleic‐acid, inorganic, and hybrid platforms, as exemplified by the following systems. i) Zn^2+^‐dependent deoxyribozymes that ligate RNA with observed rate constants up to k_obs_ ≈ 0.5 min^−1^ at 1 mM Zn^2+^ (23 °C, pH 7.9) and that form native 3′–5′ linkages—defining a minimal, DNA‐only ligase‐like catalyst.^[^ ^106^ ^]^ ii) An enzyme‐free DNA‐templated click ligation chain reaction catalyzed by heterogeneous Cu_2_O that achieves minute‐scale ligation and attomolar‐range limits of detection in integrated electrochemical/colorimetric sensors—direct evidence that a designed nano‐interface can substitute key ligase functions for ultrasensitive assays.^[^ ^107^ ^]^ iii) Functionalized gold nanoparticles that catalyze peptide ligation under mild, aqueous‐compatible conditions, illustrating how metal surfaces plus organic ligands constitute a ligase‐like microenvironment for amide‐bond formation.^[^ ^108^ ^]^ Collectively, these studies delineate a ligase‐like nanozyme framework wherein interface templating, metal coordination, and nanoconfinement cooperate to afford quantitatively efficient covalent coupling. This capability enables programmable nucleic‐acid assembly, peptide engineering, and signal amplification in biosensing.

### 2.4 Strategic Approaches for Nanozyme Enhancement

Improving nanozyme performance hinges on electronic structure–guided electron transfer, well‐defined active‐site architectures, and interfacial reaction kinetics clarified by theory‐experiment synergy. This section systematically examines advanced strategies for enhancing nanozyme efficiency through multidisciplinary approaches combining materials design with theoretical and experimental investigations.^[^ ^109^ ^]^

#### 2.4.1 Electron Transfer Pathway Optimization

Optimizing electron‐transfer (ET) pathways is pivotal for enhancing nanozyme performance, as charge separation, intermediate stabilization, and barrier crossing constitute ET‐limited steps in most catalytic cycles. Three principal strategies have been developed to address these bottlenecks: heterojunction engineering, electron mediator incorporation, and active‐site electronic structure modulation.

Heterojunction engineering creates built‐in electric fields and directional electron channels that suppress charge recombination while preserving redox capacity. Representative Z‐scheme systems such as g‐C_3_N_4_/iron‐oxide and g‐C_3_N_4_/TiO_2_ heterostructures exhibit enhanced redox capability and higher photoelectrochemical sensitivity in sensing applications. These improvements are attributed to band alignment, as confirmed by density functional theory (DFT) calculations and photophysical characterization, which enables efficient interface‐controlled carrier extraction.^[^ ^110^, ^111^ ^]^

Electron mediator incorporation facilitates interfacial electron shuttling through molecular or π‐conjugated motifs. For example, in the hemin‐MoS_2_ van der Waals hybrid, hemin serves as an electron‐transfer mediator while simultaneously inducing a partial phase transition in MoS_2_ from semiconducting (2H) to metallic (1T). This structural transformation enhances the hybrid's electrical conductivity, which directly accelerates electron transfer during catalysis and explains its superior peroxidase‐like activity toward TMB/H_2_O_2_ compared to the individual components.^[^ ^112^ ^]^

In electroanalytical contexts, CeO_2_‐decorated laser‐induced graphene (LIG) electrodes increase electrochemically active area (from 1.31 to 3.35 cm^2^) and lower charge‐transfer resistance to ≈ 762 Ω, enabling highly sensitive dopamine assays—direct evidence that mediator‐like oxides can serve as ET bridges on conductive scaffolds.^[^ ^113^ ^]^ A third, orthogonal route focuses on redox/potential matching at atomically defined sites. Fe–N–C SANs designed with bio‐inspired coordination (e.g., Fe–N_3_) exhibit up to 3.3‐fold higher OXD‐like activity than Fe–N_4_ and 8,791‐fold versus Fe_3_O_4_. DFT calculations attribute these gains to optimal adsorption energies and lowered electron transfer barriers for peroxide‐derived intermediates, a principle recently leveraged through atom‐pair engineering to further tune the local electronic structure and speed H_2_O_2_ activation.^[^ ^67^, ^114^ ^]^

Active‐site electronic structure modulation serves as a third strategic approach by optimizing energy alignment at atomically defined active sites. For instance, in reactive oxygen species regulation, carbon‐dot‐based superoxide dismutase nanozymes achieve catalytic efficiency comparable to natural enzymes. Mechanistic studies combined with DFT calculations identify that vacancy and edge states align redox potentials to facilitate O_2_•^−^ adsorption and electron‐transfer‐mediated dismutation, reaffirming energy‐level matching as a critical factor in ET‐limited processes.^[^ ^115^ ^]^

Collectively, these three mechanisms, including heterojunction‐guided charge separation, mediator‐facilitated shuttling, and active‐site potential matching, provide synergistic levers to overcome electron‐transfer bottlenecks. This integrated strategy yields higher catalytic turnover, improved selectivity, and enhanced signal transduction across photoelectrochemical and electrochemical applications.

#### 2.4.2 Active Site Precision Engineering

The rational design of active sites plays a fundamental role in advancing nanozyme functionality by establishing molecular‐level control over electronic configuration and local geometry. These structural parameters directly govern intermediate binding energetics, reorganization dynamics, and the determination of rate‐limiting steps in catalytic cycles. Three primary design strategies have emerged to achieve this precision: atomic site construction, coordination sphere optimization, and bio‐inspired cofactor integration.

Atomic site construction enables the creation of well‐defined single metal centers, maximizing site uniformity and atom economy while allowing programmable control over primary coordination environments. In a related system, single copper atoms are anchored on graphene oxide, where each copper atom is bonded to four nitrogen atoms in a square planar arrangement. This precise structure allows the material to efficiently and selectively break down superoxide radicals, even under challenging conditions. Its performance demonstrates that controlling the immediate atomic environment of the metal center is key to managing electron and proton transfer, which is crucial for controlling reactive oxygen species.^[^ ^115^ ^]^

Coordination sphere optimization provides a approach to modulate selectivity‐determining steps by controlling the polarity and binding configurations of oxygenated intermediates. In cobalt porphyrin frameworks, enhanced axial ligand fields or strategically positioned cationic groups rebalance the relative binding strengths of *OOH and *O species while enforcing specific end‐on versus side‐on adsorption geometries. These structural modifications preferentially stabilize the complete 4e^−^/4H^+^ oxygen reduction pathway over competing 2e^−^ processes. Systematic kinetic and spectroscopic analyses confirm that these precisely engineered axial and second‐sphere coordination effects constitute the fundamental mechanism for the observed selectivity enhancement.^[^ ^116^, ^117^ ^]^

Bio‐inspired cofactor integration embeds functional prosthetic groups that serve as both catalytic centers and microenvironment organizers, going beyond simple electron‐shuttling roles. For example, hemin incorporated into metal–organic frameworks exhibits enhanced peroxidase‐like activity with superior operational stability and mass transport compared to free hemin. Under optimized conditions, the TMB/H_2_O_2_ system demonstrates a linear response from 5.0×10^−6^ to 2.0×10^−4^ M (R^2^ ≈ 0.994), confirming that scaffold‐confined cofactors enable precise performance control.^[^ ^116^, ^117^ ^]^

In parallel, noble‐metal nano‐interfaces and flavin motifs reproduce essential steps of oxidase catalysis: Au nanoparticles follow a glucose‐oxidase‐like two‐step mechanism (substrate dehydrogenation followed by two‐electron O_2_ reduction to H_2_O_2_), while FAD functionalized onto Au nanostructures establishes organized electron‐transfer pathways that elevate heterogeneous ET rates and analytical sensitivity in glucose sensing.^[^ ^118^ ^]^

Taken together, these approaches establish precisely engineered catalytic architectures that emulate the sophisticated microenvironment control exhibited by natural enzymes. Through atomic site construction, coordination sphere optimization, and bio‐inspired cofactor integration, researchers have substantially expanded the functional capabilities and application potential of nanozyme technologies across diverse sensing platforms.

#### 2.4.3 Interfacial Reaction Kinetics Adjustment

Optimizing interfacial reaction kinetics focuses on the dynamic processes occurring at the solid‐liquid interface, aiming to accelerate substrate delivery, stabilize transition states, and promote product release. Three key approaches have been established to achieve these objectives: surface chemistry modification, morphological engineering, and confined microenvironment construction.

Surface chemistry modification tailors binding affinity and local concentration through molecular‐level interface design. For iron oxide nanozymes, polydopamine coatings provide catechol and amine functional groups that concentrate aromatic or anionic probes under acidic conditions while stabilizing co‐catalysts, thereby enhancing peroxidase‐like signals in colorimetric assays. These polymer shells have been successfully employed to immobilize recognition elements on Fe_3_O_4_ and to construct Fe_3_O_4_@polydopamine/gold hybrid catalysts with reinforced interfacial reactivity.^[^ ^119^, ^120^ ^]^

Mesoporous carbons with programmed hydrophobic/hydrophilic domains concentrate hydrophobic substrates (e.g., TMB) at catalytic sites while maintaining aqueous diffusion through hydrophilic channels. This architecture correlates with enhanced POD‐like activity in hierarchical, heteroatom‐doped carbons for antioxidant/biothiol assays.^[^ ^121^ ^]^

Morphological engineering addresses diffusion limitations by creating continuous transport pathways and readily accessible active sites. 3D conductive scaffolds effectively demonstrate this principle. For example, graphene and graphene oxide aerogels that support noble‐metal nanoparticles create interconnected networks, allowing simultaneous substrate diffusion and electron transport. As shown in nanoporous platinum/graphene oxide microelectrodes, these scaffolds achieve significantly higher non‐enzymatic glucose sensing sensitivity than dispersed nanoparticle systems.^[^ ^122^ ^]^ Likewise, hierarchical MoS_2_ “nanoflowers” furnish radially oriented petal edges and interlayer channels that augment POD‐like catalysis and enable rapid biothiol quantification (e.g., GSH) in colorimetric/electrochemical formats.^[^ ^123^, ^124^ ^]^

Microenvironment modulation enhances catalytic selectivity by creating confined spaces that act as molecular filters. Metal–organic frameworks with tailored pore sizes and structures can selectively admit target molecules while blocking larger interferents. This size‐ and shape‐based selection, combined with specific host–guest interactions, significantly improves detection selectivity and accelerates apparent reaction rates.^[^ ^125^, ^126^ ^]^ Complementarily, thermo‐responsive polymer brushes grafted on catalytic surfaces undergo reversible coil–globule transitions around the lower critical solution temperature (LCST) and thus modulate substrate access to active sites in situ, providing an external handle to tune activity and stability without altering the inorganic core.^[^ ^127^ ^]^

Together, synergistic integration of these approaches establishes comprehensive kinetic control: surface chemistry governs molecular recognition, morphology regulates transport efficiency, and confined environments provide selective activation. This integrated approach establishes efficient reaction microenvironments that significantly enhance catalytic performance across biosensing applications.

#### 2.4.4 Mechanistic Investigation Through Advanced Characterization

Advanced characterization techniques provide crucial insights into nanozyme catalytic mechanisms, enabling rational design and performance optimization through three primary approaches: electronic structure analysis, reaction pathway tracking, and dynamic interface monitoring.

Synchrotron‐based X‐ray absorption spectroscopy (XAS) combined with DFT calculations provides critical insights into the coordination environment and electronic states of catalytic centers.^[^ ^128^ ^]^ Methodologically, recent operando XAS overviews detail how near‐edge (XANES) and extended region (EXAFS) track redox transients and coordination changes under reaction conditions, offering guidelines for quantitative fitting and artifact control.^[^ ^129^ ^]^ A representative application involves Fe–N–C single‐atom nanozymes, where operando XAS directly observes the dynamic interconversion between Fe^2+^ and Fe^3+^ oxidation states during H_2_O_2_ activation. Complementary DFT calculations demonstrate that the well‐defined Fe–N_4_ coordination structure facilitates this redox process by optimizing electron distribution around the iron center, thereby stabilizing key reactive intermediates.^[^ ^130^ ^]^

Reaction pathway tracking with time‐resolved spectroscopy captures transient intermediates and identifies key steps in nanozyme catalytic cycles. In CeO_2_‐based systems, femtosecond transient absorption spectroscopy directly monitors charge carrier dynamics at oxygen vacancy sites, while DFT calculations reveal how these vacancies stabilize reactive oxygen species.^[^ ^131^ ^]^ Complementary vibrational techniques provide molecular‐level insights through surface‐enhanced Raman spectroscopy (SERS), which tracks reaction pathways in real time and enables precise quantification of POD‐like activity by directly observing TMB oxidation intermediates.^[^ ^132^ ^]^ Although most Fe–N–C evidence stems from electrocatalytic studies, in situ/operando Raman on Fe–N–C frameworks demonstrates how ligand/second‐sphere perturbations bias high‐valent Fe═O formation and subsequent O–O or C–H activation, offering transferable insights into OXD/POD‐mimetic routes.^[^ ^133^ ^]^

Dynamic interface monitoring combines microscopy and electrochemical techniques to visualize mass and charge transport processes at the solid‐liquid interface. Correlative electrochemical atomic force microscopy (AFM) platforms operate under working conditions to quantitatively map local conductivity, friction, and reactivity with nanoscale resolution. This approach reveals significant spatial heterogeneity while identifying reconstructed active sites that substantially lower reaction barriers. These experimental observations are well supported by DFT calculations that confirm the enhanced catalytic activity of the newly formed interfacial structures.^[^ ^134^ ^]^

Together, these coupled experimental–computational approaches convert phenomenological nanozyme activity into mechanism‐anchored design rules by identifying the operative redox states, enumerating transient surface intermediates, and quantifying structure dynamics under realistic conditions.^[^ ^135^ ^]^ The synergistic combination of advanced material design with theoretical computations and in situ spectroscopic techniques provides a powerful framework for developing next‐generation nanozymes with tailored catalytic properties for specific applications.

## 3 Nanozyme‐Biochip System

Biochips are miniaturized analytical devices that integrate biological recognition units with physical or chemical transducers to achieve high‐throughput and automated detection. When combined with nanozymes, they not only enhance catalytic amplification but also enable rapid signal responsiveness. According to the characteristics of signal presentation, nanozyme‐biochip systems can be broadly categorized into four representative modalities: electrochemical, colorimetric, fluorescent, and chemiluminescent.

In electrochemical systems, nanozymes are typically immobilized on modified electrodes to catalyze redox reactions and amplify charge‐transfer signals. Colorimetric systems utilize chromogenic or POD‐like reactions to generate visible color changes, while fluorescent systems rely on Förster resonance energy transfer (FRET) or emission quenching to modulate optical intensity. Chemiluminescent systems, on the other hand, employ luminol or co‐reactant oxidation to produce light emission without external excitation. Each subsequent subsection focuses on these distinctive mechanisms and discusses how the coupling of nanozyme catalysis and chip engineering enhances overall sensing performance.

### 3.1 Electrochemical Nanozyme‐Biochip System

**Figure 5:**
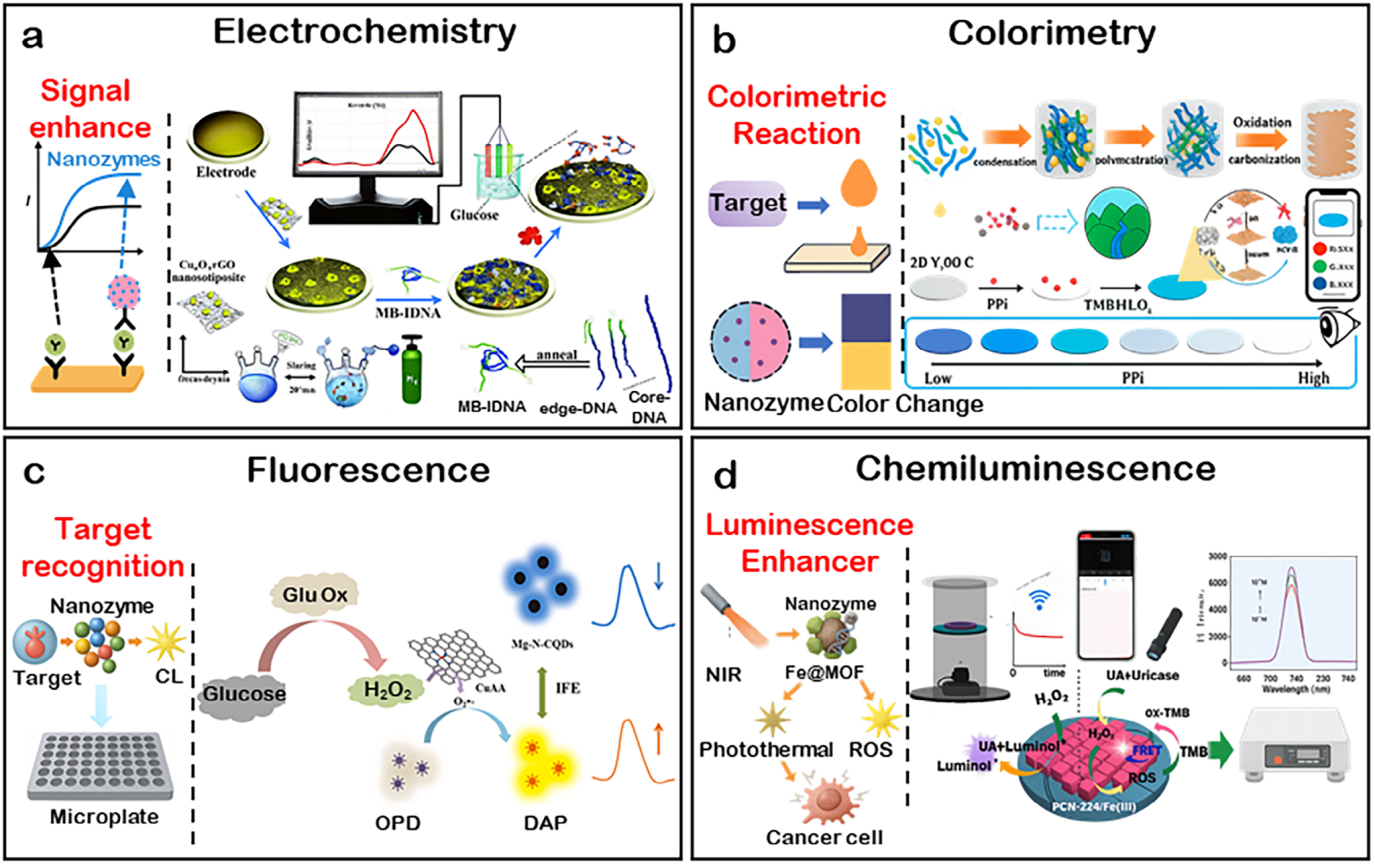
Modalities of signal responsiveness in nanozyme‐biochip systems. a) Nanozyme‐assisted signal amplification for electrochemical sensitivity enhancement. Reproduced with permission.^[^ ^137^ ^]^ Copyright 2021 Royal Society of Chemistry. b) Colorimetric sensing through POD‐mimicking nanozyme reactions. Reproduced with permission.^[142^^]^ Copyright 2024 Elsevier. c) Fluorescence target recognition via nanozyme‐coupled cascades. Reproduced with permission.^[^ ^153^ ^]^ Copyright 2022 Elsevier. d) Luminescence enhancement by nanozyme‐mediated synergistic effects. Reproduced with permission.^[^ ^157^ ^]^ Copyright 2024 Elsevier.

Electrochemical biochips represent a type of extensively investigated platform for nanozyme integration, owing to their inherent sensitivity, cost‐effectiveness, and compatibility with miniaturized devices.^[^ ^136^ ^]^ As illustrated in **Figure** **5**a, nanozyme‐biochip systems operate by immobilizing nanozymes onto conductive electrodes or porous scaffolds, where catalytic reactions convert into electrical signals, including current, potential, or impedance. The common reaction pathways involve POD‐like activation of H_2_O_2_ to generate electroactive intermediates, OXD‐like O_2_ reduction to produce H_2_O_2_ in situ, or cascade processes with signal amplification.^[^ ^137^ ^]^

In the POD‐like pathway, nanozymes catalyze the decomposition of H_2_O_2_ into electroactive intermediates that yield quantifiable current responses. Li et al. developed a ratiometric electrochemical sensor based on Cu_2_O–reduced graphene oxide nanozymes immobilized on screen‐printed electrodes, where the nanozymes produced redox currents proportional to glycated albumin concentration. Such systems can be miniaturized into biochip formats, where nanozyme‐modified electrodes allow on‐chip signal acquisition and wireless readout. These works demonstrate the transition from conventional electrodes to integrated electrochemical biochips, enhancing portability and clinical utility.

In the OXD‐like mode, nanozymes directly utilize dissolved oxygen to produce H_2_O_2_ in situ, eliminating the need for external peroxide addition. Chen et al. reported an Au–Ag modified black phosphorus nanozyme prepared by a sonoelectrochemical exfoliation method, which could be directly deposited on electrode surfaces.^[^ ^138^ ^]^ By enabling in situ H_2_O_2_ generation, the device achieved highly sensitive detection of 4‐nitrophenol with simplified operation, illustrating the potential of nanozyme‐biochip system for portable monitoring.

A cascade nanozyme system integrates OXD‐ and POD‐like activities, enabling to generate and decompose H_2_O_2_ under hyperthermal conditions.^[^ ^139^ ^]^ For example, a nanozyme system that combined MXene nanosheets with Pt nanoparticles obtained amplified signals for cancer biomarker assays while maintaining performance in complex serum matrices. At the system level, Mahmudunnabi et al. combined nanozymes with immunoassays, nucleic acid assays, and cell‐based assays in a microfluidic format, demonstrating that cascade catalysis can be harnessed for high‐throughput and reproducible detection in real biological samples.^[^ ^140^ ^]^

Taken together, these examples demonstrate that POD‐like, OXD‐like, and cascade processes provide complementary approaches for electrochemical nanozyme‐biochip construction. Nevertheless, common bottlenecks remain, including instability of immobilized nanozymes, electrode fouling in complex media, and limited selectivity for specific analytes. Emerging approaches such as advanced surface chemistries for immobilization, antifouling hybrid interfaces, and single‐atom catalysts offer promising solutions to these challenges and are expected to further enhance the translational potential of electrochemical nanozyme‐biochip systems.

### 3.2 Colorimetric Nanozyme‐Biochip System

Colorimetric biochips exploit the catalytic ability of nanozymes to oxidize chromogenic substrates, thereby generating visible colorimetric signals that can be directly interpreted by the naked eye or recorded using portable readout devices such as smartphones. As illustrated in Figure 5b, nanozymes are typically embedded within microfluidic or paper‐based platforms, where substrate oxidation induces quantifiable changes in absorbance. Owing to their operational simplicity and minimal instrumentation requirements, colorimetric systems are particularly suited for point‐of‐care diagnostics and field‐deployable testing scenarios.^[^ ^141^, ^142^, ^143^ ^]^

The main class of colorimetric systems relies on the chromogenic substrate TMB, where nanozymes mimic POD activity to catalyze substrate oxidation in the presence of H_2_O_2_. Based on this principle, Han et al. developed a gold nanozyme‐based paper analytical device for the detection of Hg^2+^ ions. In this system, AuNPs form Au‐Hg amalgams in the presence of mercury, which significantly enhances their POD‐like activity toward the catalytic oxidation of TMB by H_2_O_2_.^[^ ^144^ ^]^ By embedding nanozyme catalytic zones within paper‐based biochips, smartphone‐assisted imaging can be directly applied for on‐chip quantification. However, the requirement for peroxide poses challenges due to its inherent toxicity and instability. To overcome this limitation, Wu et al. designed a MnO_2_ nanosheet‐based platform that utilized ambient O_2_ as the terminal oxidant, eliminating the need for exogenous H_2_O_2_ and enabling selective detection of organophosphate pesticides in a portable colorimetric format.^[^ ^145^ ^]^ These representative studies demonstrate that colorimetric assays are evolving into chip‐level diagnostic tools with low cost and high accessibility.

Substrate catalysis combined with sequence‐specific hybridization is also developed for target recognition. Komkova et al. synthesized Prussian Blue‐based nanozymes via a catalytic assembly strategy and functionalized them with oligonucleotides through click chemistry, thus coupling catalytic activity with molecular recognition. This design significantly amplified the colorimetric signal and allowed ultra‐sensitive detection of DNA sequences in serum at concentrations several orders of magnitude lower than conventional assays.^[^ ^146^ ^]^

In addition to conventional chromogenic substrates, colorimetric systems have also been extended through functional materials such as molecularly imprinted polymers (MIPs). Lu et al. reported a MoS_2_/PEDOT‐based MIP nanozyme sensor tailored to recognize quercetin, a bioactive flavonoid. The molecularly imprinted polymer enhanced both binding affinity and selectivity toward the target. Subsequent catalytic oxidation of quercetin produced electrochemical and colorimetric signal outputs, demonstrating strong consistency between molecular recognition and signal readout.^[^ ^147^ ^]^

Taken together, these examples define the operational principles and implementation strategies of colorimetric nanozyme‐biochip systems. While notable advances have improved sensitivity and portability, these platforms remain constrained by environmental interference, instability of chromogenic reactions, and limited linear response ranges. Emerging strategies offer viable paths toward overcoming these limitations by integrating nanozymes with a biochip for localized environmental control or with machine learning for signal correction.

### 3.3 Fluorescent Nanozyme‐Biochip System

Fluorescence‐based biochips utilize the capacity of nanozymes to modulate signal change through catalytic generation or quenching of fluorophores. As illustrated in Figure 5c, fluorescence signals are typically regulated via three mechanisms: i) substrate transformation that releases or activates fluorescent moieties, ii) production of ROS that oxidize or quench fluorescent probes, and iii) nanozyme‐mediated FRET or inner‐filter effects (IFE) that alter emission intensity or wavelength. These versatile mechanisms provide high sensitivity and spectral selectivity, making fluorescence systems particularly suitable for detecting low‐abundance biomarkers in complex biological environments.^[^ ^141^, ^148^, ^149^ ^]^

In the category of substrate transformation, the confined catalytic geometry ensured localized signal amplification, highlighting that spatial restriction can effectively translate molecular recognition into amplified optical outputs. For example, the target‐triggered Prussian Blue nanoparticles within TiO_2_ nanochannels catalyzed the oxidation of TMB, generating charged intermediates that enhanced fluorescence.^[^ ^150^ ^]^ These fluorescence probes are incorporated within biochip architectures, ensuring precise excitation control and real‐time on‐chip readout. This mechanism enables the detection of telomerase activity in urine samples from bladder cancer patients.

For ROS‐mediated catalysis, a multi‐analyte fluorescent platform enabled simultaneous detection of ppGpp and nicotinamide adenine dinucleotide phosphate (NADPH) by integrating nanozyme catalysis with substrate‐specific probes. The system achieved rapid responses within 50 s across plant, bacterial, and serum matrices by orchestrating sequential reactions within microfluidic chambers.^[^ ^151^ ^]^ This result demonstrates the strength of cascade catalysis for multiplexed analysis.

In FRET/IFE‐based regulation, a ternary Fe_3_O_4_/MnO_2_/CeO_2_ composite with multiple enzyme‐mimetic activities was immobilized on chip surfaces and coupled to dual fluorescent probes, producing proportional ratiometric signals for ultrasensitive alkaline phosphatase detection in serum.^[^ ^152^ ^]^ Similarly, Cu‐doped amorphous carbon was combined with carbon quantum dots to trigger cascade reactions and the IFE for the detection of blood glucose. Fluorescence readouts could be directly acquired by portable optics, achieving clinically precise measurements.^[^ ^153^ ^]^ These examples collectively emphasize that ratio metric and wavelength‐shifted outputs enhance robustness against environmental fluctuations, further diversifying the functional space of fluorescent biochips.

Overall, fluorescent nanozyme‐biochip systems offer excellent sensitivity and versatility. Despite these advances, they encounter three major challenges. First, background autofluorescence from biological matrices often obscures probe signals and compromises detection limits. Second, the instability of fluorescent substrates and the spatial dependence of energy transfer hinder reproducibility, particularly under dynamic microfluidic conditions. Third, the portability of fluorescence readouts remains restricted by reliance on bulky excitation sources and detectors. Their practical translation hinges on overcoming these intrinsic challenges through rational probe design, optimized chip architectures, and portable readout integration.

### 3.4 Chemiluminescent Nanozyme‐Biochip System

Chemiluminescence (CL)‐based biochips exploit the catalytic activity of nanozymes to initiate or accelerate light‐emitting reactions, thereby generating self‐sustained optical signals with intrinsically low background noise. As illustrated in Figure 5d, these systems commonly rely on POD‐like nanozymes to catalyze luminol‐H_2_O_2_ reactions. Depending on the catalytic mechanism and implementation strategy, CL nanozyme platforms can be broadly categorized into two modes: i) H_2_O_2_‐driven systems relying on externally supplied oxidants, and ii) in situ H_2_O_2_ generation for self‐sufficient luminescence.^[155]^

In conventional H_2_O_2_‐driven systems, for example, cobalt‐doped carbon dot nanozymes are encapsulated within porous metal‐organic frameworks (Co‐CD/PMOF) to catalyze luminol‐H_2_O_2_ reactions, yielding long‐lasting luminescence and enabling aflatoxin B1 detection at levels as low as 0.217 ng mL^−1^.^[^ ^154^ ^]^ This highlights the high sensitivity achievable with external oxidants, but also reflects their dependence on reagent stability. To address this limitation, in situ H_2_O_2_ generation strategies have been explored. For example, embedding nanozymes as anodic catalysts within a biofuel cell architecture enables localized electrochemical reactions to continuously generate H_2_O_2_, thereby stabilizing luminol oxidation and supporting robust signal readout.^[^ ^155^ ^]^ These autonomous designs improve reproducibility and reduce reliance on unstable external reagents.

In addition, material and structural engineering provide additional means to regulate emission kinetics. A trimetallic AuPtCo nanozyme is constructed to slow substrate diffusion, which can extend emission duration and produce a steady “glow‐type” signal suitable for reproducible biomarker detection.^[^ ^156^ ^]^ Similarly, Ni/Co‐MOF nanozymes provide abundant catalytic sites and a high surface area, which amplify signal outputs and enable ultralow‐level detection of florfenicol at 0.033 pg mL^−1^.^[^ ^157^ ^]^ When embedded into microfluidic biochip channels, these composites enable portable and high‐throughput electrochemiluminescence (ECL) assays. Furthermore, spatial confinement strategies have been explored, as illustrated by the co‐encapsulation of nanozymes and natural enzymes within hollow mesoporous silica nanochannels, achieving sustained luminescence for more than 12 h and facilitating real‐time, long‐term monitoring applications.^[^ ^158^ ^]^ Collectively, these examples demonstrate that material and structural designs can prolong emission and enhance robustness, expanding the functional space of CL‐based biochips.

In summary, chemiluminescent nanozyme‐biochip systems are progressing from simple luminol‐oxidation platforms toward multifunctional sensing systems that integrate catalytic regulation, emission control, and autonomous operation. Their convergence with flexible substrates, wireless signal transmission, and AI‐assisted data analysis is expected to establish CL nanozyme‐biochip systems as practical tools for biosensing. To provide a concise comparison of the diverse detection modalities discussed above, we summarizes the representative signal mechanisms, transduction principles, and analytical performances of nanozyme‐biochip systems based on different readout strategies (**Table** **1**).

**Table 1:** Comparison of representative signal transduction modalities in nanozyme‐biochip systems.

Detection modality	Signal mechanism	Typical detection target	Detection range / LOD	Key features	Refs.
Electrochemistry	Nanozyme‐catalyzed redox reaction on modified electrodes; electron‐transfer amplification	Glucose; H_2_O_2_; PSA	10^−9^–10^−6^ M LOD <10^−10^ M	High sensitivity Suitable for miniaturized integration	[136, 137, 138, 139, 140]
Colorimetry	Chromogenic / POD‐like oxidation generating visible color change	H_2_O_2_; Glucose; Pathogens	10^−8^–10^−5^ M LOD ≈ 10^−9^ M	Simple and visual readout Portable Low‐cost	[141, 142, 143, 144, 145, 146, 147]
Fluorescence	FRET regulation or fluorescence quenching between nanozyme and fluorophore	DNA; Proteins; Metal ions	10^−11^–10^−8^ M LOD ≈ 10^−12^ M	Ultra‐sensitive Multiplexed spectral analysis	[141, 148, 149, 150, 151, 152, 153]
Chemiluminescence	Luminol or co‐reactant oxidation producing light emission	ROS; Enzymes	10^−10^–10^−7^ M LOD ≈ 10^−12^ M	No external excitation Low background noise	[154, 155, 156, 157, 158]

## 4 AI‐Integrated Nanozyme‐Biochip System

**Figure 6:**
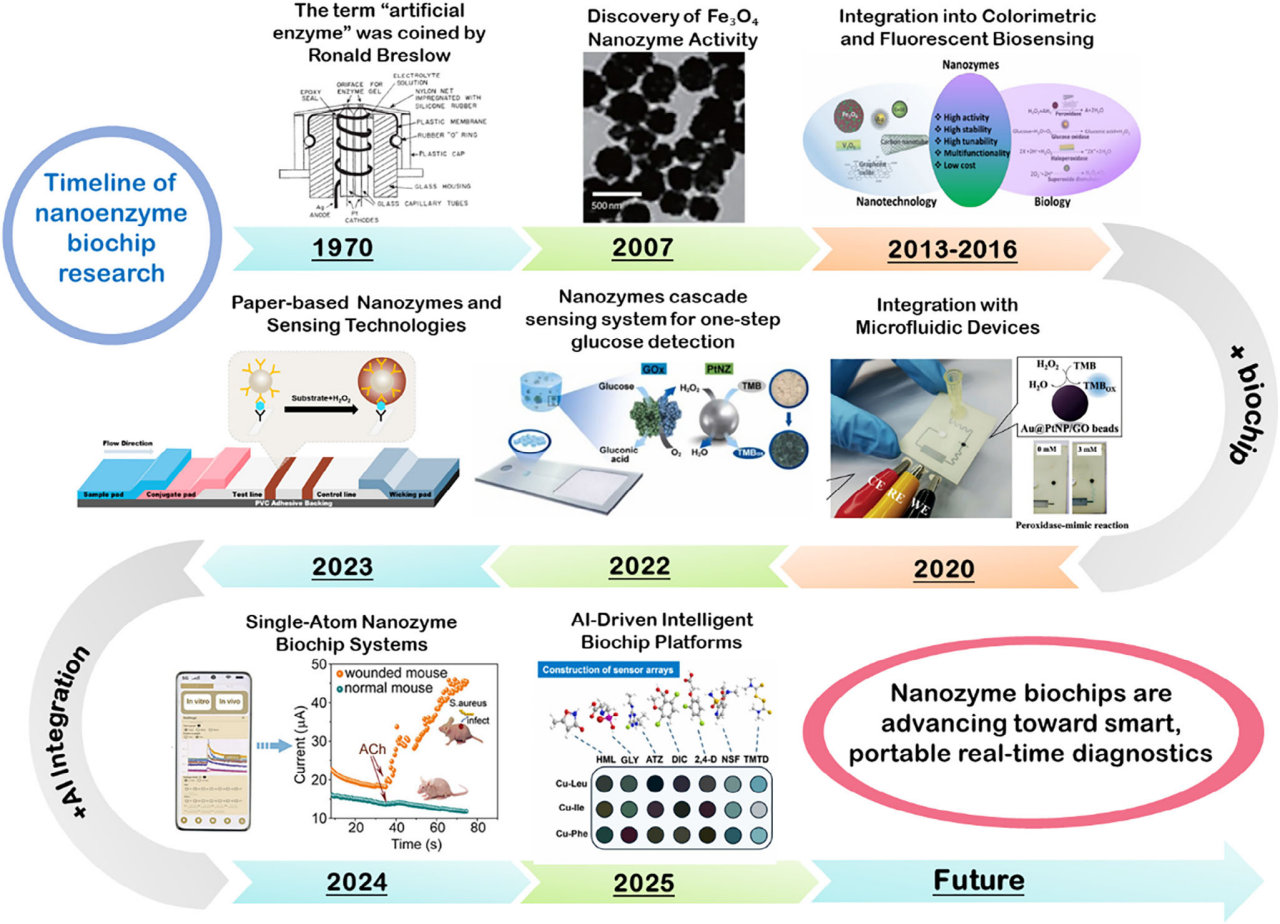
Timeline of nanozyme‐biochip system, highlighting the transition from individual nanozyme research to AI‐integrated intelligent biosensing systems. Reproduced with permission. Copyright 1967 Springer Nature, 2007 Springer Nature, 2016 Springer Nature, 2023 MDPI, 2022 Elsevier, 2019 Elsevier, 2023 American Chemical Society, 2025 Elsevier.

AI excels at complex data processing and intelligent decision‐making, enabling transformative capabilities. However, conventional nanozyme‐biochips often struggle with reproducibility and adaptability in complex detection scenarios. The integration of AI with nanozyme‐biochip systems has propelled their evolution from isolated signal enhancers into high‐throughput, high‐precision, and adaptive sensing systems. This trajectory can be delineated into three developmental stages (**Figure** **6**):

- Initiation stage (1970–2016). The seminal discovery that Fe_3_O_4_ nanoparticles exhibit intrinsic POD‐like activity positioned nanozymes as robust alternatives to natural enzymes. In this stage, their applications were primarily confined to colorimetric and fluorescence‐based assays, where they enhanced sensitivity and reduced costs in established techniques such as polymerase chain reaction (PCR) and enzyme‐linked immunosorbent assay (ELISA).^[^ ^159^ ^]^ While these early studies validated the feasibility of nanozymes, their role was largely limited to single‐signal amplification without integration into broader analytical frameworks.
- Expansion stage (2016–2023). The convergence of nanozymes with microfluidic platforms enabled the emergence of portable point‐of‐care testing (POCT) devices, bridging laboratory innovations with practical diagnostic scenarios.^[^ ^160^ ^]^ Simultaneously, rational material design extended their applications into multimodal imaging, underscoring its versatility in precision medicine.^[^ ^161^ ^]^ Despite these advances, signal readout and interpretation remained dependent on manual analysis, constraining system adaptability under complex or variable conditions.
- Intelligent stage (2023‐Future). Recently, the combination of wearable nanozyme sensors with AI‐driven feedback has established the basis for personalized and context‐aware diagnostics.^[^ ^162^ ^]^ Beyond algorithmic innovation, this stage emphasizes the co‐optimization of hardware and software, where sensing modules, power management, and computing architectures are designed as a synergistic system, which lays the groundwork for intelligent biosensing.

Across these stages, AI is gradually providing the computational means to interpret and integrate information, thereby becoming an essential element for transforming nanozyme‐based sensors into practical and intelligent platforms. Based on this emerging trend, the following discussion highlights the main algorithms and their primary applications in driving progress in nanozyme‐biochip systems: i) decoding complex signals, ii) classifying multiple analytes, and iii) integrating multimodal data.

### 4.1 AI in Nanozyme‐Biochip Signal Analysis

AI technologies have become crucial for processing complex signals in nanozyme‐biochip systems, enabling automated interpretation of multi‐modal data with high accuracy and efficiency. These algorithms transform raw sensor outputs into reliable diagnostic information through advanced pattern recognition and real‐time processing capabilities. According to their methodological features, AI algorithms used in nanozyme‐biochip systems can be broadly categorized into four families (**Table** **2**).

**Table 2:** Representative AI algorithms and their applications in nanozyme‐biochip systems, illustrating sensing modality, application scenario, and comparative advantages.

Category	Specific Algorithms	Key Metric	Data Analysis Applications	Advantages	Limitations	Refs.
Deep Learning	CNN, RNN, LSTM, Transformer	Accuracy: 94–98% Inference Speed: 50–200ms Training Data: 10K‐100K samples Model Size: 50–500MB	CNN: Spatial feature extraction from colorimetric signals RNN/LSTM: Time‐series analysis of electrochemical kinetics Transformer: Multi‐spectral data fusion and analysis	High accuracy with complex signals End‐to‐end processing capability Robust to signal variations	High computational requirements ‐ Large training data needed ‐ Black‐box nature limits interpretability	[163, 164, 165, 166, 167, 168, 169]
Computer Vision	YOLO, U‐Net, ResNet	mAP: 0.89‐0.96 IoU: 0.85‐0.93 Processing Speed: 10–100ms/image Detection Accuracy: 92–97%	YOLO: Real‐time detection zone localization U‐Net: Precise signal region segmentation ResNet: Signal intensity classification and background subtraction	Fast processing speed High spatial resolution Robust to illumination changes	Sensitive to image quality Requires calibration for different devices Limited to visual signal types	[170, 171, 172]
Machine Learning	XGBoost, SVM, Random Forest	Accuracy: 85–93% Training Time: 1–10 min Feature Dimensions: 20–100 Cross‐validation Score: 0.82‐0.90	XGBoost: Regression analysis for concentration quantification SVM: Signal pattern classification and interference detection Random Forest: Multi‐parameter signal correlation analysis	Works with small datasets Model interpretability Fast training and inference	Requires manual feature engineering Limited to structured data Performance plateaus with complex patterns	[173, 174, 175, 176]
Edge AI	TensorFlow Lite, TinyML, ONNX Runtime	Model Size: 1–10MB Power Consumption: 0.5‐2W Latency: 10–50ms Memory Usage: 10–100MB	Real‐time sensor data processing on mobile devices Low‐latency signal analysis for point‐of‐care use Power‐efficient continuous monitoring	Enables portable deployment Low energy consumption Offline operation capability	Reduced model accuracy Limited model complexity Hardware dependency issues	[162, 177, 178, 179, 180]

#### 4.1.1 Deep Learning Algorithms

Deep learning algorithms serve critical functions in processing complex signal patterns in nanozyme‐biochip systems. Among them, convolutional neural networks (CNNs) excel in extracting spatial features from colorimetric signals and microscopic images, while recurrent neural networks (RNNs) and long short‐term memorys (LSTMs) capture temporal dependencies in electrochemical reaction kinetics.^[^ ^163^, ^164^ ^]^ Transformers handle multi‐modal data integration, combining spectroscopic, electrochemical, and optical signals for comprehensive analysis.^[^ ^165^ ^]^ During the process of algorithm construction, dataset involves collecting 10,000‐100,000 annotated samples through automated signal acquisition systems, with data augmentation techniques including noise injection, time‐warping, and spectral transformation.^[^ ^166^ ^]^ Training employs a 80‐10‐10 split for training‐validation‐test sets, using transfer learning from pre‐trained models and adversarial training for domain adaptation.^[^ ^167^, ^168^ ^]^ Validation strategies incorporate k‐fold cross‐validation, temporal hold‐out validation for time‐series data, and performance monitoring on out‐of‐distribution samples to ensure model robustness.^[^ ^163^, ^169^ ^]^

#### 4.1.2 Computer Vision Algorithms

Computer vision algorithms specialize in visual signal processing and analysis within nanozyme‐biochip platforms. YOLO enables real‐time detection and localization of reaction zones in microfluidic chips, U‐Net provides precise segmentation of colorimetric signal regions, and ResNet classifies signal intensity levels while eliminating background interference.^[^ ^170^ ^]^ During this process of algorithm construction, dataset requires 5000–20 000 high‐resolution images captured under standardized lighting conditions, with annotations including bounding boxes for detection zones and pixel‐level masks for signal regions.^[^ ^171^ ^]^ Data augmentation employs geometric transformations, color space adjustments, and synthetic defect generation.^[^ ^166^ ^]^ Training utilizes progressive image resolution scaling and multi‐scale training techniques, with validation through precision‐recall metrics, intersection‐over‐union (IoU) scoring, and clinical expert verification to ensure diagnostic‐grade accuracy.^[^ ^172^ ^]^

#### 4.1.3 Machine Learning Algorithms

Traditional machine learning algorithms provide efficient solutions for structured signal processing tasks in nanozyme‐biochip systems. XGBoost performs regression analysis for concentration quantification, SVM classifies signal patterns to distinguish specific from non‐specific binding, and Random Forest identifies feature importance in multi‐parameter sensor data.^[^ ^173^, ^174^ ^]^ During this process of algorithm construction, dataset focuses on feature‐engineered data from 500–2000 experimental runs, incorporating material properties, environmental conditions, and signal characteristics.^[^ ^175^ ^]^ Feature selection employs recursive feature elimination and mutual information scoring.^[^ ^176^ ^]^ Training implements nested cross‐validation with hyperparameter optimization via grid search and Bayesian methods.^[^ ^166^ ^]^ Validation strategies include leave‐one‐batch‐out cross‐validation to assess batch‐to‐batch consistency and permutation tests to verify feature significance, ensuring model reliability across different manufacturing lots.^[^ ^176^ ^]^

#### 4.1.4 Edge AI Algorithms

Edge AI algorithms enable real‐time signal processing in resource‐constrained nanozyme‐biochip deployments. TensorFlow Lite optimizes pre‐trained models for mobile inference, TinyML develops lightweight architectures for microcontroller deployment, and ONNX Runtime ensures cross‐platform compatibility.^[^ ^177^, ^178^ ^]^ Dataset construction of edge AI algorithms involves hardware‐in‐the‐loop collection from target devices, capturing performance metrics under varying power conditions and processing constraints.^[^ ^162^, ^179^ ^]^ Training employs neural architecture search to balance accuracy and efficiency, coupled with quantization‐aware training for 8‐bit integer deployment.^[^ ^165^ ^]^ Knowledge distillation transfers learning from larger teacher models to compact student networks.^[^ ^180^ ^]^ Validation incorporates latency profiling, power consumption monitoring, and thermal performance testing under continuous operation, with additional stress testing for memory usage and computational load to guarantee reliable field deployment.^[^ ^178^ ^]^

Recognizing these methodological distinctions is essential for rationally selecting algorithms that balance accuracy, interpretability, and computational efficiency in future nanozyme‐biochip developments.

### 4.2 AI for Interpreting Complex Signal in Nanozyme‐Biochip Systems

Nanozyme‐biochip systems generate intrinsically complex signals because nanozymes possess diverse catalytic pathways and heterogeneous active sites (e.g., defect/single‐atom centers, mixed valence states). These chemical features lead to non‐linear kinetics and multi‐dimensional readouts, such as electrochemical peak shifts, multi‐channel colorimetric responses, and fluorescence spectral variations, that are difficult to interpret directly under realistic noise and drift. Within this context, AI serves not simply as a generic analytics tool but as a means to decode nanozyme‐specific signal complexity and convert it into reliable quantitative outputs on chip.

**Figure 7:**
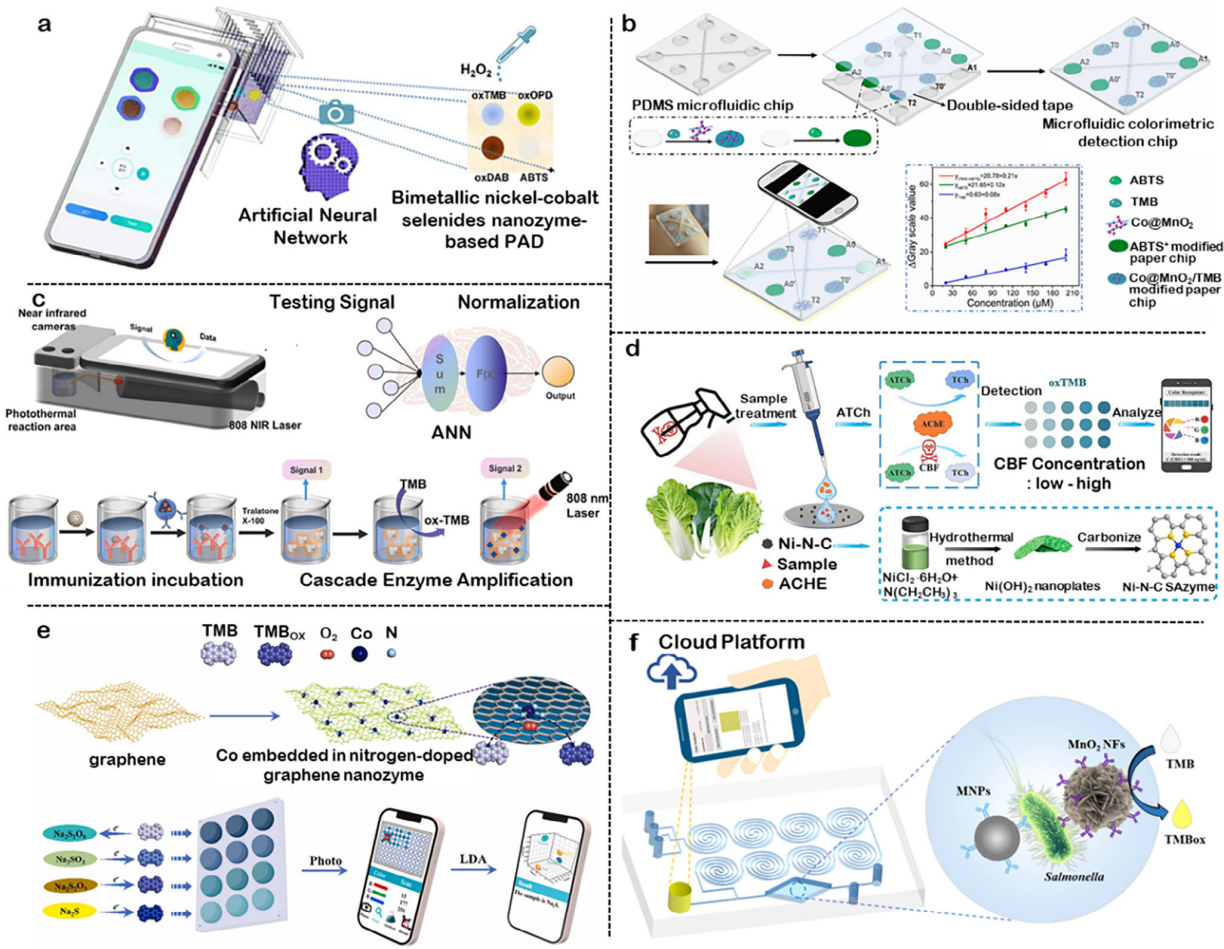
AI‐assisted nanozyme‐biochip systems for intelligent sensing. a) An AI‐powered paper‐based nanozyme‐biochip systems enabling multi‐channel detection of H_2_O_2._ Reproduced with permission.^[^ ^182^ ^]^ Copyright 2024 Elsevier. b) A smartphone‐interfaced colorimetric microfluidic chip utilizing cobalt‐doped MnO_2_ nanozymes for visual H_2_O_2_ detection. Reproduced with permission.^[^ ^135^ ^]^ Copyright 2024 Elsevier. c) A machine learning‐based nanozyme array for rapid diagnosis of urinary tract infections. Reproduced with permission.^[^ ^183^ ^]^ Copyright 2022 Elsevier. d) A smartphone‐readable nanozyme system for pesticide residue analysis via acetylcholinesterase inhibition and oxTMB signal output. Reproduced with permission.^[^ ^188^ ^]^ Copyright 2025 Elsevier. e) A single‐atom cobalt nanozyme array combined with linear discriminant analysis for classification of sulfur‐containing salts. Reproduced with permission.^[^ ^192^ ^]^ Copyright 2023 Elsevier. f) A cloud‐connected MnO_2_ nanozyme microarray enabling smartphone‐based *Salmonella* detection for real‐time result sharing. Reproduced with permission.^[^ ^194^ ^]^ Copyright 2021 American Chemical Society.

One important dimension of this progress concerns feature extraction from weak nanozyme signals. In a violet phosphorus‐porous carbon nanozyme‐based biochip system, machine learning discriminated subtle catalytic peak‐current shifts from baseline noise, achieving near‐perfect accuracy (R^2^ = 0.9999) by targeting features directly rooted in nanozyme reaction kinetics.^[^ ^181^ ^]^ On another paper‐based nanozyme‐biochip system, AI decoded multi‐channel H_2_O_2_ outputs (e.g., cross‐channel intensity patterns rather than a single absorbance value), offering a representative example of intelligent interpretation in low‐signal settings (**Figure** **7**a).^[^ ^182^ ^]^ These studies clearly show that AI can uncover weak signals that are almost invisible to human interpretation.

Another key aspect lies in calibration and drift compensation associated with nanozyme variability. Batch‐to‐batch differences in catalytic activity and environmental illumination introduce biases that conventional calibration cannot remove. Embedding an artificial neural network (ANN) to learn RGB ratios and grayscale gradients corrected illumination inconsistencies while preserving the underlying nanozyme reaction contrast on paper chips (Figure 7b).^[^ ^182^ ^]^ Likewise, by jointly analyzing colorimetric intensity with the slope of concomitant temperature trajectories, a three‐layer ANN differentiated cardiac troponin I concentrations down to 10.8 pg mL^−1^, demonstrating that coupling orthogonal readouts generated during nanozyme reactions improves clinical‐grade precision.^[^ ^183^ ^]^

A further advance is seen in interpretable learning for complex spectral outputs of nanozyme reactions. CNNs applied to Raman spectra highlight which bands‐product/intermediate‐linked peaks versus baseline‐drive decisions, aligning model saliency with nanozyme‐catalyzed chemistries.^[^ ^184^ ^]^ Explainable methods (e.g., Grad‐CAM, attention) further localize diagnostic bands that most contribute to classification, thereby improving interpretability and aiding mechanism‐aware optimization of the nanozyme‐biochip interface.^[^ ^185^ ^]^

Taken together, AI critically enhances nanozyme‐biochip systems by confronting their core challenges: it extracts subtle features from weak catalytic signals, compensates for variability and drift intrinsic to nanozyme reactions, and parses complex spectral information in a mechanistically interpretable manner. This integrated framework establishes a powerful synergy wherein the biochip provides controlled, multiplexed transduction pathways, while AI decodes the informational richness embedded within the nanozyme's catalytic kinetics. This partnership constitutes the critical factor driving these systems toward robustness and real‐time application in smart detection.

### 4.3 AI for Classifying Multiple Analytes in Nanozyme‐Biochip Systems

Analyte classification requires distinguishing different species or subtypes within mixed samples, a task that becomes particularly challenging when signals overlap or fluctuate under real‐world conditions. Nanozymes, owing to their tunable catalytic activity, generate highly diverse signal fingerprints, such as distinct colorimetric intensities, electrochemical impedance profiles, or spectral features. When transduced through biochip platforms, these heterogeneous catalytic signatures provide a rich but complex dataset. AI offers a powerful route to decode and classify these nanozyme‐derived fingerprints, transforming catalytic diversity into reliable categorical outputs.

A representative direction is pathogen classification using nanozyme‐based arrays. Yang et al. designed a Fe–N–C nanozyme colorimetric array chip where machine learning algorithms distinguished subtle variatons in intensity patterns and cross‐well absorbance, achieving 97% accuracy in identifying multiple urinary tract infection (UTI) pathogens (Figure 7c).^[^ ^186^ ^]^ Here, the ability to generate differential catalytic fingerprints from a single array of nanozymes directly provided the substrate for AI‐based classification.

A further illustration comes from food safety monitoring using nanozyme electrodes. Zhu et al. embedded ANN algorithms into MoS_2_/MWCNT‐based nanozyme electrodes, enabling real‐time classification of pesticide residues in tea and rice by analyzing catalytic fingerprints reflected as peak potential shifts and current density variations.^[^ ^187^ ^]^ Similarly, Zhang et al. developed a smartphone‐readable nanozyme‐biochip where AI interpreted acetylcholinesterase‐inhibition/oxTMB outputs to rapidly classify pesticide residues in complex food matrices (Figure 7d).^[^ ^188^ ^]^ These studies demonstrate that AI enables real‐time and on‐site decoding of catalytic signatures that nanozymes inherently generate during biochemical reactions.

More advanced developments involve subtype discrimination based on spectral and electrochemical responses of nanozyme systems. Zhang et al. applied CNNs to identify electrochemical impedance spectra, where the model learned features such as Nyquist semicircle shapes and phase differences that are tightly linked to nanozyme‐catalyzed interfacial processes.^[^ ^189^ ^]^ Likewise, Liu et al. demonstrated that deep CNNs could directly interpret raw Raman spectra by extracting subtle peak variations without preprocessing to achieve robust molecular subtype classification.^[^ ^190^ ^]^ These approaches emphasize that nanozyme‐generated fingerprints span both electrical and optical domains, and AI is able to distill these heterogeneous features into robust diagnostic categories.

In summary, the integration of AI with nanozyme‐biochip systems creates a powerful paradigm for analyte classification that transcends the limitations of conventional sensing. This synergy establishes a cohesive workflow: the tunable catalytic properties of nanozymes generate a rich library of distinct signal fingerprints, which are then standardized through a biochip platform for multiplexed acquisition. Subsequently, AI algorithms serve as the computational engine to decode these complex, overlapping patterns into accurate categorical decisions. By effectively harnessing catalytic diversity as an information‐rich resource, this integrated approach enables robust and multiplexed classification across a growing spectrum of real‐world applications, from clinical to on‐site food and environmental monitoring.

### 4.4 AI‐Integrated Multimodal Biosensing with Nanozyme‐Biochip Systems

Beyond interpreting individual signals and classifying different analytes, recent research has increasingly emphasized the importance of integrating multiple heterogeneous signals into unified outputs. This approach is particularly valuable when single modalities are weak or unstable, since complementary information from multiple channels can be combined to produce more reliable results. However, significant interference may occur among different detection modalities. For example, electrochemical processes may alter local pH or generate species that quench fluorescent signals. Colorimetric products can absorb light at wavelengths used for fluorescence excitation or emission, causing inner‐filter effects. Simultaneous detection can lead to crosstalk, where a single analyte triggers responses in multiple channels.^[^ ^153^ ^]^

To address these challenges, several effective approaches have been developed in nanozyme‐biochip systems. For instance, spatial separation of detection zones within microfluidic chips to physically isolate different sensing reactions.^[^ ^148^ ^]^ Temporal sequencing of measurements to ensure signals are acquired in non‐interfering time windows.^[^ ^158^ ^]^ When differentiation through the aforementioned experimental approaches proves challenging, advanced computational algorithms offer a powerful alternative. For instance, computational methods include multivariate calibration and machine learning algorithms that mathematically disentangle overlapping signals using pre‐established interference models.^[^ ^190^ ^]^

An early example of this concept is demonstrated by Zhang et al., who reported a trimodal platform integrating ELISA, ECL, and light‐emitting diode (LED) imaging. Machine learning was used to merge image‐derived texture and intensity features with biomarker concentrations, leading to enhanced diagnostic accuracy than any single assay alone.^[^ ^183^ ^]^ This demonstrates that the implementation of AI algorithms facilitates more precise detection of target analytes through decoding of multimodal signals.

Building on this foundation, researchers have begun to apply signal integration strategies directly to nanozyme‐biochip systems. Yu et al. coupled a Prussian Blue nanozyme‐biochip system with a smartphone interface, using an ANN algorithm to simultaneously interpret colorimetric intensity and temperature variations.^[183,^ ^191^ ^]^ By coordinating optical and thermal trajectories, the model maintains robust detection performance even under fluctuating environmental conditions. Similarly, researchers employed single‐atom cobalt nanozyme arrays that generated multiple response dimensions (including signal intensity, kinetic profiles, and spectral variations). By integrating these heterogeneous signal features with a statistical method such as linear discriminant analysis (LDA), they successfully classified different sulfur‐containing salts(Figure 7e). This approach demonstrated that even relatively simple statistical frameworks can benefit from multi‐signal fusion, underscoring the versatility of nanozyme‐biochip systems in complex analyte discrimination.^[^ ^192^ ^]^

Further advances extend to quantitative biosensing and imaging. In imaging, Conesa et al. developed an AI algorithm to merge fluorescence and Raman outputs, combining morphological and chemical cues to surpass the resolution of either modality alone.^[^ ^193^ ^]^ For imaging applications, Conesa et al. developed an AI algorithm that integrates fluorescence and Raman data. This fusion of morphological and chemical information enables superior resolution, exceeding the limits of each individual modality.^[^ ^194^ ^]^ Although not all such approaches directly involve nanozymes, the paradigm enables to be directly translated to nanozyme‐based imaging, where catalytic processes generate distinct signal signatures (Figure 7f).^[^ ^195^ ^]^

Taken together, AI‐integrated multimodal biosensor enhances nanozyme‐biochip systems by leveraging nanozymes as the source of multiple signal channels, using biochips to capture these channels in parallel, and employing AI to unify and interpret multimodal signals. This strategy adeptly turns multimodal complexity into a cornerstone for developing robust, interpretable, and practical biosensing platforms across multiple fields (clinical, food, and environmental scenarios).

## 5 Application of Nanozyme‐Biochip System

The convergence of nano‐catalysis, biochip engineering, and AI signal processing is poised to revolutionize practical biosensing. This transformative potential is already being actualized in diverse application scenarios. To concretely illustrate this translational pathway, we present a critical examination of their deployment in three key areas: clinical diagnostics, food safety, and environmental monitoring.

### 5.1 Disease Diagnosis

Timely disease diagnosis is crucial for guiding clinical decision‐making and facilitating personalized treatment. Although traditional analytical technologies have made progress in disease monitoring, they still face challenges in sensitivity, specificity, and operability within complex clinical settings. The nanozyme‐biochip systems enable to enhance detection sensitivity, multiplexity, and robustness for comprehensive monitoring from early screening to therapeutic efficacy assessment.^[^ ^196^ ^]^ This synergistic approach has been employed to improve the technical performance for detecting multiple diseases, such as diabetes, Alzheimer's disease (AD), cardiovascular diseases, infectious diseases, and tumors.

#### 5.1.1 Diabetes

Diabetes may lead to severe complications, including cardiovascular disorders, retinopathy, and diabetic foot ulcers, which significantly compromise both physical health and quality of life. Accurate monitoring of blood glucose levels is essential for diabetes management.^[^ ^197^, ^198^ ^]^ Conventional assays face critical limitations, including the intrinsic instability of natural enzymes under physiological conditions and the requirement for bulky optical/electrochemical instrumentation.

Nanozyme‐biochip systems overcome these barriers by mimicking the catalytic activity of natural enzyme while integrating miniaturized transduction systems for point‐of‐care testing.^[^ ^199^ ^]^ Among these, the glucose oxidase (GOx)‐like activity of gold nanoparticles has been extensively utilized for detection of glucose.^[^ ^200^ ^]^ For instance, Arshad et al. developed a paper‐based microfluidic device incorporating AuNR‐CeO_2_ nanozyme. By leveraging the GOx‐like activity of AuNR and POD‐like activity of CeO_2_ within the nanozyme, glucose in complex biological matrices can be catalytically converted into H_2_O_2_, thereby inducing the color reaction of TMB.^[^ ^201^ ^]^ This colorimetric sensor demonstrates a linear response across 0.5–500 mg dL^−1^ with a limit of detection (LOD) of 0.65 mg dL^−1^. Additionally, this system showed result‐agreement rates of 92% and 88% compared to two commercial products, while also demonstrating superior stability over traditional enzymatic assays by maintaining performance for over 4 weeks at 4°C.

**Figure 8:**
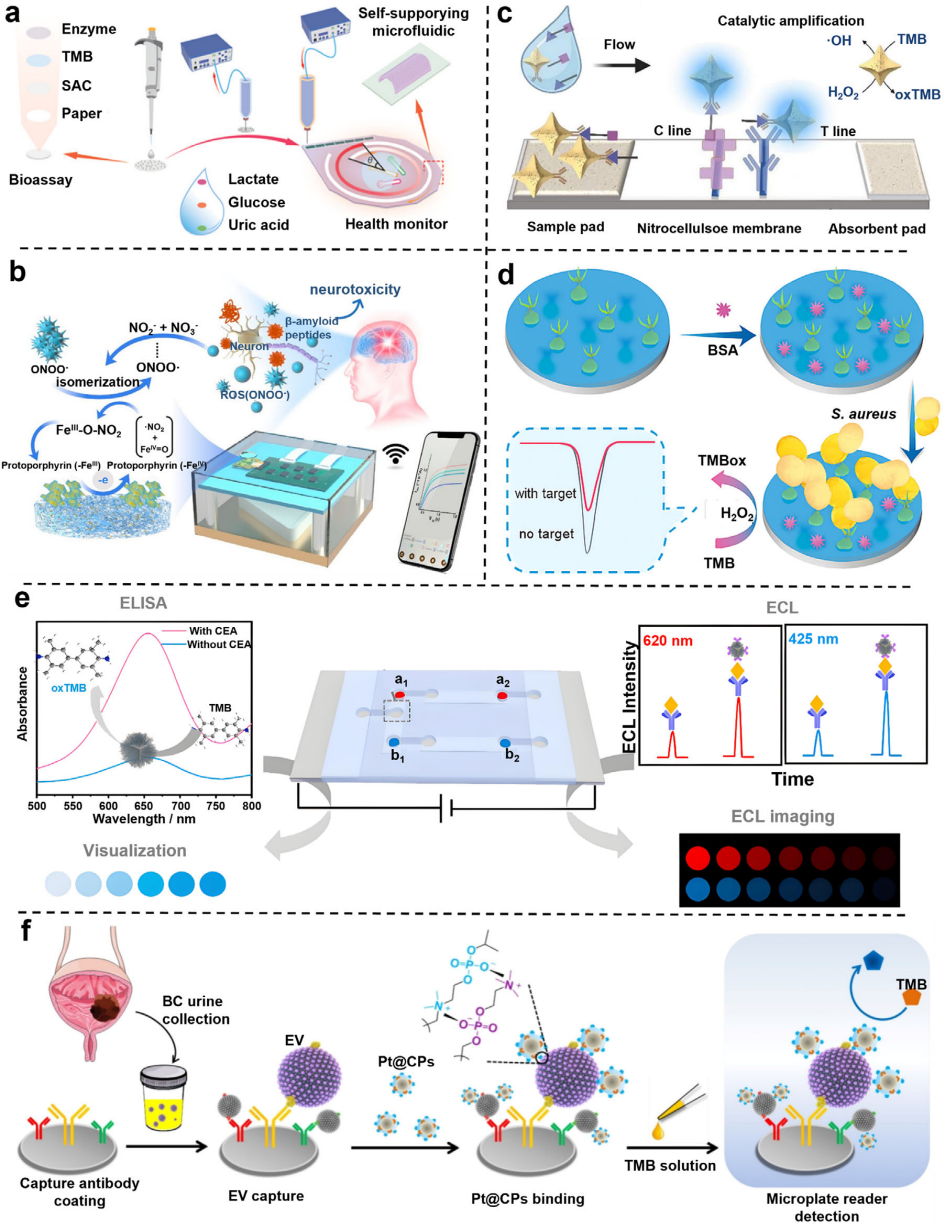
Nanozyme‐biochip systems for disease diagnostic applications. a) Flexible wearable health monitor incorporating nanozymes for real‐time detection of glucose, lactate and uric acid levels in sweat. Reproduced with permission.^[^ ^202^ ^]^ Copyright 2024 American Chemical Society. b) Portable sensing platform combined with nano‐enzymatic detection of ONOO^−^. Reproduced with permission.^[^ ^213^ ^]^ Copyright 2023 American Chemical Society. c) Nanozymes‐conjugated biosensor for the detection of cTnI by colorimetric lateral chromatography assay (CLFA). Reproduced with permission.^[^ ^218^ ^]^ Copyright 2025 Elsevier Ltd. d) Phage nano‐enzymatic binding sensors for specific detection of *S. aureus*. Reproduced with permission.^[^ ^225^ ^]^ Copyright 2024 Elsevier Ltd. e) Nanozymes combined with biochips to detect CEA. Reproduced with permission.^[^ ^234^ ^]^ Copyright 2024 Elsevier Ltd. f). High‐sensitivity detection of BC biomarkers MUC‐1, CCDC25, and GLUT1 using platinum nanozyme on biochips. Reproduced with permission.^[^ ^239^ ^]^ Copyright 2024 American Chemical Society.

Advancing toward real‐time monitoring of blood glucose, Chen et al. reported a 3D‐printed flexible epidermal patch embedded with Fe–N–C SANs (**Figure** **8**a).^[^ ^202^ ^]^ This system collects sweat through a skin‐attached microchannel, which directs the fluid into the integrated colorimetric biosensor module of the nanozyme‐biochip system. Within this module, the sweat reacts with specific oxidases and chromogens to produce color changes. Images are then captured via a smartphone camera and processed through color calibration algorithms to quantitatively analyze the concentrations of glucose, lactate, and uric acid in sweat. Subsequent analysis indicates that the glucose and lactate detected by this flexible patch exhibit higher sensitivity compared to commercial enzyme‐based detection methods. The system provides real‐time physiological feedback, enabling personalized metabolic profiling and preventive healthcare strategies.

#### 5.1.2 Alzheimer's Disease

AD is the most common neurodegenerative disease worldwide, affecting an estimated 57.4 million people in 2019 and the number projected to increase to 152.8 million by 2050.^[^ ^203^ ^]^ Current clinical diagnostic methods primarily include positron emission tomography (PET) for visualizing regional distribution in brain, and lumbar puncture for analyzing β‐amyloid (Aβ) and phosphorylated tau protein in cerebrospinal fluid. However, these methods remain limited by high cost, invasive procedures, as well as safety concerns.^[^ ^204^, ^205^ ^]^

Nanozyme‐biochip systems enable highly sensitive detection of ultra‐low concentrations of AD biomarkers in blood.^[^ ^206^, ^207^, ^208^, ^209^ ^]^ For example, Zhang et al. developed a nanozyme‐catalyzed colorimetric sensor for rapid, highly sensitive detection of Aβ42 protein (a type of Aβ) within neuron‐derived extracellular vesicles (EVs) in plasma. This strategy employs Fe_3_O_4_@SiO_2_ core‐shell nanoparticles for magnetic capture of EVs and utilizes the POD‐like activity of Au@Pt nanomaterials to catalyze a colorimetric reaction with TMB substrate, enabling specific detection of Aβ42‐positive EVs.^[^ ^210^ ^]^ Comparative clinical validation against standard ELISA showed that the sensor achieved an area under the curve (AUC) of 1.0, while ELISA yielded an AUC of 0.8889, indicating superior diagnostic accuracy. By mimicking natural enzymatic activities and combining with microfluidic technology, this system simplifies the operation process and provides a non‐invasive, low‐cost approach for AD diagnosis.

Beyond using Aβ42 as biomarker, recent research indicates that the dysregulation of peroxynitrite (ONOO^−^) homeostasis precedes Aβ deposition, offering a promising avenue for early screening and prevention of AD.^[^ ^211^, ^212^ ^]^ As shown in Figure 8b, Peng et al. developed a highly sensitive multi‐engineered graphene extended‐gate field‐effect transistor (EG‐FET) sensor for detecting ONOO^−^ production in brain tissue.^[^ ^213^ ^]^ Significantly enhanced electrochemical performance and selectivity (with LOD as low as 0.02 nM) were achieved through the synergistic combination of laser‐induced graphene (LIG) with MnO_2_ nanoparticles and GO‐MnO_2_/Hemin nanozymes. The EG‐FET sensor is further integrated into a microfluidic chip, enabling rapid and accurate monitoring through automatic sampling of microliter‐level samples. Detection signals are transmitted in real time to a smartphone app via the built‐in Bluetooth module. Leveraging robust data processing capabilities and a visual interface, the app not only displays real‐time concentration‐response curves and historical trends but also supports cloud storage and remote data sharing. This provides a portable, efficient solution for early AD screening and dynamic medical warning.

#### 5.1.3 Cardiovascular Disease

Cardiovascular disease (CVD), including acute myocardial infarction (AMI) and heart failure (HF), has become the leading cause of global mortality and disability, with prevalence continues to rise, especially in the context of an aging population.^[^ ^214^, ^215^ ^]^ Early diagnosis and dynamic monitoring of CVD biomarkers are crucial for improving prognosis. Traditional detection technologies (e.g., ELISA, PCR) have bottlenecks such as equipment dependency, long testing periods, and high costs, which limits their use in primary healthcare organizations and home self‐testing. Therefore, the development of highly sensitive POCT technologies is of great significance for enhancing disease prevention and control.

For the early diagnosis of AMI, cardiac troponin I (cTnI) and microRNA‐133a, as the “gold standard” biomarkers, offer the advantages of high myocardial specificity.^[^ ^216^, ^217^ ^]^ Jiang et al. developed a PtIr@Fe‐MOF nanozyme‐based lateral flow biosensor for ultrasensitive cTnI detection (Figure 8c). The system employs a CRISPR‐mediated signal amplification strategy, where target binding triggers isothermal amplification and subsequent cleavage of a dual‐labeled DNA reporter. The cleaved products, captured at the test line, enable PtIr@Fe‐MOF nanozymes to catalyze color development for visual and AI‐integrated quantification. This platform achieved sensitive detection of cTnI across a dynamic range of 0.01–10 pg mL^−1^, with a detection limit as low as 10 fg mL^−1^. Compared with commercial ELISA kits, quantitative analysis revealed strong linear correlation between the two methods (R^2^ = 0.9958), while demonstrating improved detection times (30 min). Furthermore, the platform was rigorously validated using 70 clinical serum samples, demonstrating high diagnostic performance with 100% clinical sensitivity and specificity.^[^ ^218^ ^]^ For detection of miRNA‐133a, Li et al. developed a photoelectrochemical‐electrochemical (PEC‐EC) dual‐mode biosensor based on photo‐nanocatalyst synergistic amplification. The sensor employs POD‐like copper‐tetraphenyl porphyrin metal‐organic framework (Cu‐TCPP) to catalyze the decomposition of H_2_O_2_ under illumination. The obtained amplified signal achieved detection limits of 0.003 fM and 0.02 fM in PEC and EC modes, respectively. In subsequent clinical sample testing, the dual‐modality detection approach demonstrated greater operational simplicity and enhanced stability over traditional qPCR. It also exhibited good recovery rates (96.5–106.0%) in human serum samples, providing a reliable platform for the early detection of AMI‐related miRNA biomarkers.^[^ ^217^ ^]^

In clinical, the diagnosis of HF mainly relies on the detection of N‐terminal B‐type natriuretic peptide precursor (NT‐proBNP).^[^ ^219^ ^]^ By combining the POD‐like V_2_(Sn_0.8_Pt_0.2_)C MAX nanozyme with lateral flow immunoassay (LFIA) platform, high‐sensitivity detection of NT‐proBNP was achieved. The nanozymes are coupled with antibodies through the EDC/NHS crosslinking method to form an immune probe.^[^ ^220^ ^]^ This probe specifically recognizes NT‐proBNP in the sample and then combines with the capture antibody on the T line during the chromatography process, forming a double antibody sandwich complex. Subsequently, by adding TMB/H_2_O_2_ chromogenic substrates, the nanozyme exhibits high POD‐like activity to catalyze the colorimetric reaction, achieving an ultralow detection limit of 0.0016 ng mL^−1^ (≈1437‐fold improvement over that of traditional gold nanoparticle LFIA (2.3 ng mL^−1^)). Simultaneously, the platform demonstrated a broad linear detection range of 0.02 to 71.64 ng mL^−1^ in real human plasma samples, covering the clinical reporting range for NT‐proBNP. By analyzing the T/C line gray ratio using mobile imaging and ImageJ software, quantitative detection of NT‐proBNP can be achieved without requiring large‐scale equipment. This approach demonstrates significant advantages in detection sensitivity, quantitative range, and point‐of‐care applicability.

#### 5.1.4 Infectious Diseases

Infectious diseases arise from pathogen infection by bacteria, viruses, fungi, or parasites and remain a major cause of global morbidity and mortality.^[^ ^221^, ^222^ ^]^ In particular, bacterial and viral infections exert a substantial disease burden and can rapidly progress if not diagnosed in time. Accurate and timely detection of these pathogens is therefore essential for clinical diagnosis and effective treatment. Nanozyme‐biochip systems integrate nanozymes as signal amplification elements with miniaturized biochip platforms, enabling ultrasensitive and rapid detection of pathogenic agents in clinical samples.

For the detection of bacteria, nanozymes can capture bacteria through van der Waals forces and electrostatic interactions, triggering signal changes, while integration with biochip platforms for rapid in situ detection.^[^ ^223^, ^224^ ^]^ However, this non‐specific recognition may compromise detection accuracy. To enhance targeting ability, Gao et al. conjugated phage SapYZU04 with MoO_3_/MIL‐125‐NH_2_ nanozymes to utilize phage tail receptor‐binding proteins (RBPs) on phage to specifically recognize wall teichoic acid (WTA) on *Staphylococcus aureus* (*S. aureus*) (Figure 8d). In this design, the phage‐modified nanomaterials selectively capture viable *S. aureus* cells from the test sample onto the electrode surface, where host‐specific recognition enables electrochemical catalysis. This system achieved sensitive and selective detection of *S. aureus*, with a linear detection range of 10^1^–10^8^ colony‐forming unit (CFU)/mL and a LOD of 16 CFU mL^−1^.^[^ ^225^ ^]^

Compared to bacterial infections, viral infections demand detection methods with higher sensitivity and specificity due to their nanoscale size, intracellular concealment, and rapid mutation rate.^[^ ^226^ ^]^ Especially, the recent COVID‐19 pandemic has further accelerated the demand for rapid viral diagnostics.^[^ ^227^ ^]^ In this context, Wu et al. developed a multiplexed immunoassay based on Au@Pt NPs nanozymes and a microfluidic chip, which can detect eight respiratory viruses simultaneously, including neo‐coronaviruses. Its features include the antibody‐specific recognition of conserved proteins to minimize cross‐reactivity, nanozyme‐enhanced signaling for amplified sensitivity, and microfluidic chip integration for high‐throughput detection. This system achieves high sensitivity as low as 0.1 pg mL^−1^ and rapid analysis within 40 min, with clinical validation demonstrating 97.2% positive agreement with reverse transcription polymerase chain reaction (RT‐PCR) results.^[^ ^228^ ^]^

In addition, multimodal signal strategies have also been employed to enhance detection accuracy. For instance, a Zr‐MOF‐loaded Ru@U6‐Ru/Pt NPs‐enhanced colorimetry and ECL dual‐mode biosensing platform has been reported for rapid and sensitive detection of monkeypox virus (MPXV). Colorimetry enables rapid on‐site screening and ECL delivers highly sensitive quantitative analysis. The platform's detection speed (less than 15 min) is faster than conventional qPCR and offers higher sensitivity (10 aM). It also provides an efficient and reliable tool for timely diagnosis of MPXV and can be extended to other pathogens.^[^ ^229^ ^]^

#### 5.1.5 Tumor

Early‐stage tumor often lacks noticeable clinical symptoms, thereby limiting the efficacy of conventional detection techniques for the timely identification of microscopic lesions.^[^ ^230^, ^231^ ^]^ In contrast, an integrated platform combining nanozyme‐catalyzed amplification with chip‐level devices enables highly sensitive detection of circulating tumor markers, providing reliable foundations for subsequent precision treatments and dynamic monitoring of disease progression.^[^ ^232^ ^]^

The core advantage of nanozyme‐biochip systems lies in the ultra‐sensitive detection of trace tumor biomarkers.^[^ ^233^ ^]^ For example, by constructing an Au@NiCo_2_O_4_@MnO_2_ nanozyme‐based biochip system, researchers achieved dual‐signal amplification for detecting CEA with a LOD of 43.65 fg mL^−1^, which is a three‐order‐of‐magnitude improvement in sensitivity over conventional ELISA (Figure 8e). For clinical validation involving 98 serum samples (48 from colorectal cancer patients and 50 healthy controls), CEA detection demonstrated a sensitivity of 96.5% and specificity of 94.2%. This technology significantly enhances the accuracy of screening for early‐stage tumor biomarkers and disease diagnosis.^[^ ^234^ ^]^

Since different tumor types express distinct tumor biomarkers, identifying these specific biomarkers enables accurate classification of tumor categories.^[^ ^235^, ^236^ ^]^ For example, alpha‐fetoprotein (AFP) is a type of liver cancer marker. A recent study has developed a direct AFP detection technology from whole blood samples through enzyme‐mediated assembly of silver nanoparticles with magnetic nanoparticles. This catalytic amplification strategy eliminates complex sample pretreatment and significantly shortens the detection process.^[^ ^237^ ^]^ Such detection system is further expanded to detect dual biomarkers, carbohydrate antigen 125 (CA125) and human epididymal protein 4 (HE4), both associated with ovarian cancer. Through the magnetic separation and ECL luminescence coupling strategy, the simultaneous analysis of the two markers in serum samples can be completed within 15 min, offering an efficient solution for clinical testing and diagnosis.^[^ ^238^ ^]^

To detect bladder cancer, Li et al. developed an innovative biosensing platform by engineering a choline phosphate‐grafted platinum nanozyme (Pt@CP) via molecular imprinting (Figure 8f). This design enables the universal labeling of urinary extracellular vesicles (EVs) through multivalent “CP‐phosphocholine” interactions with the EV membrane. In a clinical cohort of 99 individuals (48 bladder cancer, 27 cystitis, and 24 healthy donors), the platform demonstrated superior diagnostic performance by simultaneously profiling three EV protein biomarkers: mucin‐1 (MUC‐1), coiled‐coil domain containing 25 (CCDC25), and glucose transporter‐1 (GLUT1). The assay achieved an AUC of 98.3%, significantly outperforming conventional methods like urine cytology, which typically has a sensitivity of ≈56%. Furthermore, its detection limit for EVs was 38‐fold lower than that of traditional ELISA, providing direct evidence of its enhanced sensitivity. A key translational advantage is its capability for post‐operative monitoring. The assay successfully identified residual lesions in patients even when cystoscopy results were negative, highlighting its potential for guiding clinical management beyond initial diagnosis.^[^ ^239^ ^]^

In conclusion, nanozyme‐biochip systems demonstrate comprehensive advantages such as high throughput and low cost, making them particularly suitable for rapid disease diagnosis in resource‐limited areas (**Table** **3**). Capable of detecting a broad spectrum of biomarkers, ranging from metabolic small molecules to macromolecules, and from single indicators to multi‐omics integration, nanozyme‐biochip systems are poised to become a key carrier of the “sample‐in‐results‐out” diagnostic platform. Beyond clinical diagnostics, their versatility naturally extends to broader public health domains. In particular, food safety monitoring requires rapid and on‐site detection, making it a direct continuation of these biomedical applications.^[^ ^240^ ^]^

**Table 3:** Application of nanozyme‐biochip systems in disease diagnosis.

Application type	Analyte	Nanozymes	Nanomaterials	Chip features	Number of clinical samples	Detection range	LOD	Ref
Blood sugar	Glucose	POD	PtNZHG	Colorimetric	2	0.01–10 mM	3.9 µM	[241]
	Glucose Uric acid Lactate	POD	Fe–N–C SACs	Colorimetric	1	0.01–0.3 mM 1–50 mM 10–100 µM	0.038 µM 6.76 µM 6.98 µM	[202]
	Glucose	GOx, POD	CeO_2_‐AuNR	Colorimetric	1	0.5–500 mg dL^−1^	0.65 mg dL^−1^	[201]
	Glucose	GOx, POD	LAON	Chemiluminescence	6	0.5–100 µM	0.1 µM	[242]
Alzheimer's Disease	Aβ 1–40	POD	Fe‐N_x_ SANs	Colorimetric	/	1–2000 pg mL^−1^	0.88 pg mL^−1^	[206]
	Tau protein BACE1	POD	Zr‐MOF/MB/Au	Electrochemistry	20	0.01 pg/m –1000 ng mL^−1^ 0.005 pg mL^−1^–0.01 ng mL^−1^	3.34 fg/mL 1.67 fg/mL	[207]
	Tau‐441	Phosphatase	Au@CeO_2_	Fluorescence immunoassay	/	0.0005–10 ng mL^−1^	0.2 pg mL^−1^	[208]
	Tau protein	POD	MoS_2_/Au	HCR	12	0.1 pg mL^−1^–100 ng mL^−1^	33.4 fg/mL	[209]
	Aβ42	POD	Fe_3_O_4_@SiO_2_, Au@Pt	Colorimetric	20	1 × 10^5^–1 × 10^9^ particles/mL	7.2 × 10^4^ particles/mL	[210]
	ONOO^–^	Peroxynitrite isomerase‐mimic	MnO_2_/LIG	Electrochemistry	/	0.02 nM–111.11 µM	0.02 nM	[213]
Cardiovascular disease	miRNA‐133a	POD	Cu‐TCPP/BiVO_4_	PEC EC	1	0.1–1000 fM	0.003 fM 0.02 fM	[217]
	cTnI	POD	PtNPs@Cu MOF	UV detection Colorimetric	3	0.003 ng mL^−1^–10 ng mL^−1^ 0.01 ng mL^−1^–7 ng mL^−1^	2.57 pg mL^−1^ 0.013 pg mL^−1^	[216]
	cTnI	POD	PtIr@Fe‐MOF	CLFA	70	0.01–10 pg mL^−1^	10 fg/mL	[218]
	NT‐proBNP	POD	Au@PdPt	Electrochemistry	1	0.1–100 pg mL^−1^	0.046 pg mL^−1^	[219]
	NT‐proBNP	POD	V_2_(Sn_0.8_Pt_0.2_)C MAX	LFIA	5	0.02–71.64 pg mL^−1^	0.0016 ng mL^−1^	[220]
Infectious diseases	*S. enteritidis*	OXD	MnO_2_	Colorimetric	7	10^3^–10^7^ CFU mL^−1^	10^3^ CFU mL^−1^	[223]
	*S. aureus*	POD	MoO_3_/MIL‐125‐NH_2_	Electrochemistry	20	10^1^–10^8^ CFU mL^−1^	16 CFU mL^−1^	[225]
	Cariogenic bacteria	POD	Fe–N–C	Colorimetric	4	10^2^–10^7^ CFU mL^−1^	10^2^ CFU mL^−1^	[224]
	MPXV	Nuclease, POD	Ru@U6‐Ru/Pt NPs	ECL Colorimetric	50	60–3 × 10^11^ copies/µL 0.1–5 × 10^5^ pM	10 aM 0.1 pM	[229]
	Influenza A, Influenza B, RSV, SARS‐CoV‐2, HboV, HMPV, AdV, HPIV	POD	Au@Pt	Colorimetric	315	0.1–1000 pg mL^−1^	0.1 pg mL^−1^	[228]
Tumor markers	CEA	POD	Ab_2_@Au@Co_3_O_4_/CoFe_2_O_4_ HNCs	ELISA ECL	/	5 × 10^−3^–100 ng mL^−1^ 8.5 × 10^−5^–100 ng mL^−1^	1.86 pg mL^−1^ 1.48 fg/mL	[233]
	CEA	POD	Ab_2_@Au@NiCo_2_O_4_@MnO_2_	ELISA ECL	5	1 × 10^−2^–100 ng mL^−1^ 1 × 10^−4^–50 ng mL^−1^	2.5 pg mL^−1^ 43.65 fg/mL	[234]
	AFP	CAT	Ag‐MNPs_30_‐NH_2_	Immunosensor	40	2.5–160 ng mL^−1^	0.56 ng mL^−1^	[237]
	CA125 HE4	POD	PtNP	VAC	20	50–200 U/mL 1–10 ng mL^−1^	0.001 U/mL 0.1 pg mL^−1^	[238]
	EV	POD	Pt@CP	Colorimetric	108	0.1–40 µg mL^−1^	0.07 µg mL^−1^	[239]

S. enteritidis, Salmonella enteritidis. VAC, Visual amplification chip.

### 5.2 Food Testing

Beyond clinical applications, nanozyme‐biochip systems also play a vital role in safeguarding public health through food safety monitoring. Persistent challenges such as pesticide residues and toxic contaminants continue to threaten the food supply chain, highlighting the urgent need for rapid and on‐site detection technologies.^[^ ^243^, ^244^ ^]^ Conventional assays remain costly, time‐consuming, and require specialized personnel.^[^ ^245^, ^246^ ^]^ By contrast, portable nanozyme‐biochip platforms have been developed to address these limitations, enabling efficient detection of foodborne pathogens, mycotoxins, pesticide residues, and nutrients. This section systematically discusses their applications in ensuring food quality and biochemical defense.

#### 5.2.1 Foodborne Pathogen

Foodborne pathogens, such as *Salmonella*,^[^ ^247^ ^]^ *Escherichia coli* (*E. coli*),^[^ ^248^ ^]^ *Listeria monocytogenes*,^[^ ^249^ ^]^ and *S. aureus*,^[^ ^250^ ^]^ can cause acute food poisoning and even death. According to the World Health Organization (WHO), foodborne diseases result in ≈600 million cases of illness and 420,000 deaths each year. Nanozyme‐biochip systems facilitate an effective solution for detecting these pathogens, enabling source control, process monitoring, and precise traceability to enhance food safety.

**Figure 9:**
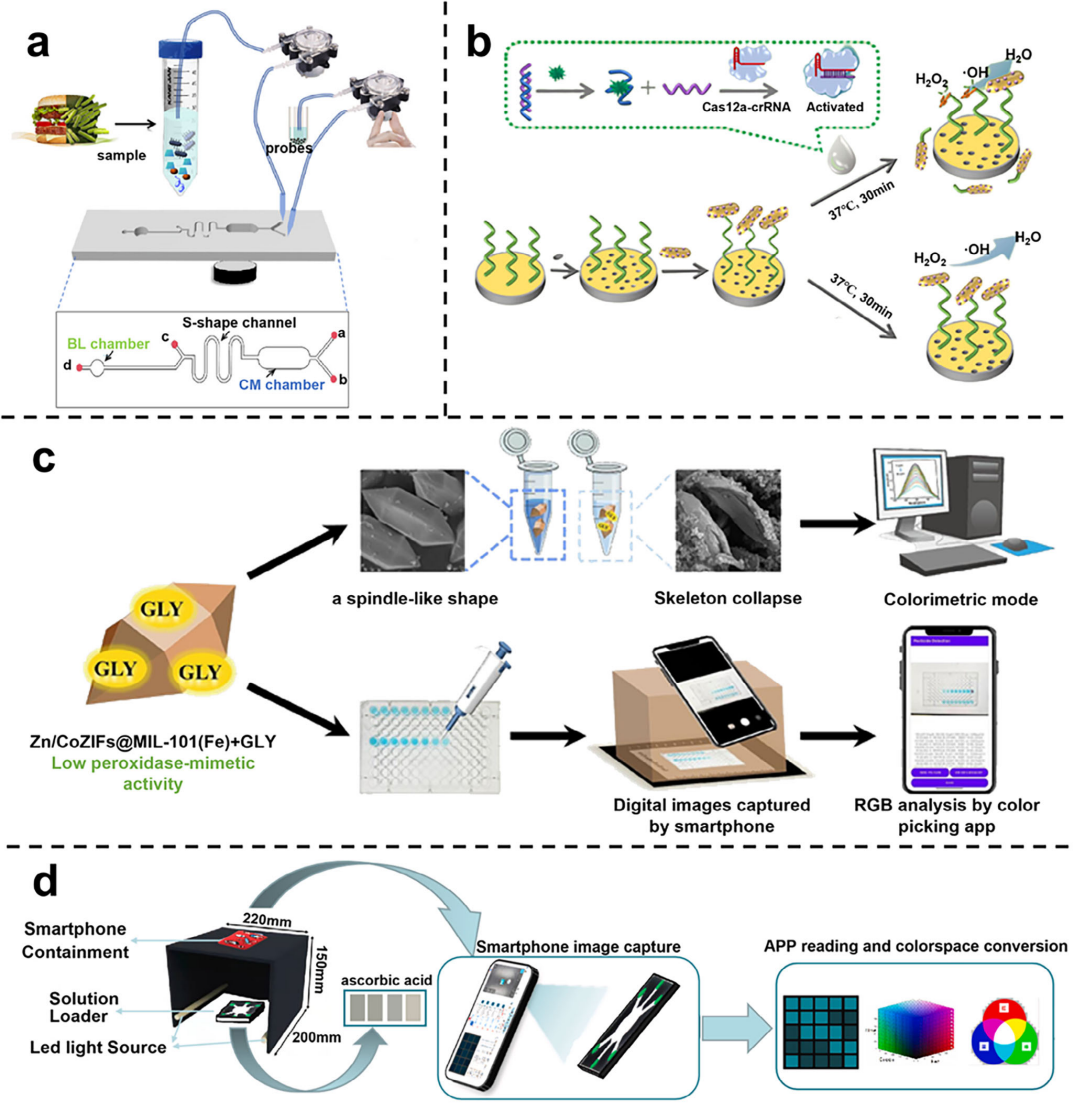
Nanozyme‐biochip systems for food testing. a) A microfluidic chip platform based on Pt‐AMP composite probes enables dual‐mode detection of *S. typhimurium*. Reproduced with permission.^[^ ^251^ ^]^ Copyright 2024 Elsevier. b) The sensitive detection of OTA by combining CRISPR technology with Pd@PCN‐222 nanozyme. Reproduced with permission.^[^ ^262^ ^]^ Copyright 2024 Elsevier. c) Colorimetric sensor based on ternary metal‐organic frame nanozyme for trace detection of GLY. Reproduced with permission.^[^ ^270^ ^]^ Copyright 2024 Elsevier. d) A highly sensitive paper‐based colorimetric system for rapid and portable detection of ascorbic acid. Reproduced with permission.^[^ ^274^ ^]^ Copyright 2023 Elsevier.

To detect foodborne pathogens, Liu et al. prepared the composite probes consisting of deactivated phage‐modified magnetic beads and platinum nanozyme‐labeled antimicrobial peptide, which can specifically recognize Gram‐negative bacteria.^[^ ^251^ ^]^ As shown in **Figure** **9**a, they also developed an integrated microfluidic chip based on dual‐mode analytical strategy to rapidly assess bacterial activity. This system allows for the replacement of phage probes to detect other bacteria, demonstrating broad potential for pathogen screening.

*Salmonella*, a widespread enteric pathogen in nature, is one of the most common causes of foodborne illness.^[^ ^252^, ^253^ ^]^ For rapid and sensitive detection of *Salmonella*, Xue et al. developed an automatic microfluidic biosensor based on MnO_2_ nanozyme.^[^ ^194^ ^]^ This system integrates MnO_2_ NFs for signal amplification, a microfluidic chip with a convergence‐divergence spiral micromixer for automatic operation, and a smartphone app equipped with a saturation‐based algorithm for image analysis. The detection process starts with immunomagnetic nanoparticles (MNPs) capturing *Salmonella*, followed by mixing with MnO_2_ NFs in the spiral micromixer to form MNP‐bacteria‐MnO_2_ sandwich complexes, which are magnetically separated in on‐chip chambers for efficient detection. This system enables the detection of *Salmonella* within 45 min with a detection limit as low as 44 CFU mL^−1^, demonstrating strong potential for on‐site detection of foodborne pathogens.

To speed up the procedure, Jiang et al. developed a colorimetric nanozyme‐biochip system.^[^ ^254^ ^]^ First, the bacterial samples, immunomagnetic nanobeads and nanozyme were rapidly mixed to form nanobead‐bacteria‐nanozyme conjugates and then magnetically separated for target enrichment. After washing, the conjugates catalyze a colorless substrate and effectively amplify signals from a blue product. Finally, the intensity is quantified using ImageJ software to determine the bacterial concentration. By utilizing finger‐operated microfluidic chips, this system enables sensitive on‐site screening for *Salmonella Typhimurium* within 25 min. These nanozyme‐biochip strategies offer a low‐cost and portable platform for on‐site detection of foodborne pathogens, thereby significantly enhancing food safety monitoring.

#### 5.2.2 Mycotoxin

Mycotoxins, such as aflatoxins, ochratoxins, fumonisins, zearalenone, and deoxynivalenol, are toxic secondary metabolites produced by fungi.^[^ ^255^ ^]^ They commonly occur in corn, peanuts, and other agricultural products. Through the food chain, these residues may accumulate in humans and cause various diseases such as kidney disease, cirrhosis, cancer, and even death.^[^ ^256^, ^257^ ^]^ Therefore, developing rapid and reliable detection methods is crucial for food safety. Indeed, nanozyme‐biochip systems offer a promising strategy for meeting this requirement.

Aflatoxins represent a prevalent form of mycotoxin, distinguished by their formidable toxicity which constitutes a grave menace to human and animal health.^[^ ^258^ ^]^ The B_1_ subtype of aflatoxin (AFB_1_) is classified as a Group I carcinogen by the International Agency for Research on Cancer.^[^ ^259^ ^]^ To achieve sensitive and portable detection of AFB_1_, Ma et al. synthesized a target‐responsive hydrogel and integrated it into a microfluidic POCT device.^[^ ^260^ ^]^ In the presence of AFB_1_, the AFB_1_ aptamer in hydrogel binds with AFB_1_, leading to the destruction of hydrogel and the release of AuNPs and platinum nanoparticles (PtNPs). The released AuNPs turn the supernatant from colorless to red, while the PtNPs catalyze H_2_O_2_ decomposition to generate O_2_. The resulting increase in gas pressure drives the movement of a red ink within the volumetric bar chart chip, providing a quantitative relationship between the distance and the concentration of AFB_1_. This method is applied to detect AFB_1_ in beer samples, achieving a detection limit of 1.77 nM—a result that complies with food safety testing standards.

Ochratoxin A (OTA), commonly present in moldy grains, can cause liver and kidney damage upon ingestion, posing serious health risks to both animals and humans.^[^ ^261^ ^]^ In a further study, Wu et al. developed a sensitive method for detecting OTA by combining a CRISPR enzyme cascade with the Pd@PCN‐222 nanozyme (Figure 9b).^[^ ^262^ ^]^ The Pd@PCN‐222 can catalyze H_2_O_2_ to produce a stable reduction peak current. When OTA is present, Cas12a becomes activated and cleaves the ssDNA labeled on Pd@PCN‐222, thereby reducing the reduction peak current of H_2_O_2_. The system attains sensitive detection of OTA (1.21 pg mL^−1^) through the measurement of changes in peak current. Furthermore, a microfluidic chip is integrated into the system to simplify and streamline the detection process, which holds great potential for on‐site applications. These nanozyme‐biochip approaches demonstrate highly sensitive and portable detection capabilities for mycotoxins such as AFB_1_ and OTA, thereby significantly advancing on‐site food safety monitoring.

#### 5.2.3 Pesticide Residue

In the course of agricultural development, the excessive use of pesticides has increased the probability of residual contamination in food.^[^ ^263^ ^]^ Since common pesticides like organophosphorus pesticides (OPs) are highly toxic, their persistence on food threatens consumer safety.^[^ ^264^ ^]^ Consequently, developing sensitive and accurate on‐site monitoring strategies is of great significance for protecting human health from the effects of pesticide residues.

Recently, nanozyme‐biochip systems have been developed for the detection of pesticide residues. For instance, Lin et al. developed a colorimetric system that integrated self‐assembly nanocomposites with a simple paper‐based test strip for on‐site detection of OPs.^[^ ^265^ ^]^ The method relies on the inhibition of acetylcholinesterase (AChE) by OPs, which prevents the original color change. The resulting color change on the paper‐based chip can be visually identified to determine the test results, offering a low‐cost solution for on‐site screening.

Similar to the OP, cypermethrin (CBF) also inhibits AChE activity. Following this principle, Zhang et al. developed a smartphone‐based colorimetric paper strip sensor to detect CBF pesticide residues.^[^ ^188^ ^]^ Briefly, Ni‐N‐C SANs exhibits highly efficient POD‐like activity for H_2_O_2_ catalysis. In the presence of AChE, the catalytic activity of Ni‐N‐C SANs is suppressed and undergoes color changes. This visible change can be accurately quantified using a smartphone system to detect the concentration of CBF. This colorimetric paper strip sensor provides a real‐time method for the detection of CBF residues in vegetables.

Beyond AChE inhibition‐based assays, the nanozyme inhibition strategy has also been widely adopted for colorimetric pesticide detection.^[^ ^266^, ^267^ ^]^ For example, Feng Li's group designed a Cu‐doped carbon nanozyme exhibiting high OXD‐like activity, whose catalytic function is specifically inhibited by agricultural fungicide thiophanate‐methyl (TM).^[^ ^268^ ^]^ This fundamental interaction enabled highly selective TM detection with a sensitivity of 0.04 µg mL^−1^. By integrating a smartphone for colorimetric result interpretation, the method enabled on‐site detection of TM in environmental samples. Building upon this foundation, the group further engineered 2D V_2_O_5_ nanosheets (2D‐VONz) with unique peroxidase‐mimetic properties.^[^ ^269^ ^]^ By incorporating these nanomaterials into paper‐based chips, they successfully extended the pesticide‐nanozyme inhibition strategy to detect herbicide glyphosate (Gly), demonstrating the methodology's versatility. In a parallel development for Gly detection, Yang's group developed a colorimetric sensing platform based on ternary metal‐organic frameworks (ZnCo‐ZIFs@MIL‐101(Fe)) (Figure 9c).^[^ ^270^ ^]^ This platform leverages the synergistic effects of multiple metals in nanozyme to significantly enhance POD‐like activity. Furthermore, it integrates paper‐based microfluidic chips and smartphone, demonstrating excellent accuracy in testing real samples such as cabbage and oranges. These studies provide comprehensive insights from nanozyme mechanisms to practical detection applications, establishing a universal translational foundation for nanozyme‐biochip systems in pesticide detection.

Gly is a widely used herbicide, and its excessive residues in food, agricultural products, water and soil pose serious threats to human health and the environment.^[^ ^271^ ^]^ For the detection of Gly, Yang's group developed a colorimetric sensing platform based on ternary metal‐organic frameworks (ZnCo‐ZIFs@MIL‐101(Fe)) (Figure 9c).^[^ ^270^ ^]^ This platform leverages the synergistic effects of multiple metals in nanozyme to significantly enhance POD‐like activity. Furthermore, it integrates paper‐based microfluidic chips and smartphone, demonstrating excellent accuracy in testing real samples such as cabbage and oranges. This novel methodology demonstrates significant potential for real‐time monitoring of food safety.

#### 5.2.4 Nutrient

Beyond clinical applications, nanozyme‐biochip systems have also been developed for food safety monitoring, addressing contamination issues that threaten population‐wide health. Nutrients in food form the essential foundation to maintain physiological functions and overall health. Adequate nutrition can enhance immune function, whereas excessive intake may cause harm to the body.^[^ ^272^ ^]^ Therefore, the accurate detection of nutrients in food is vital for human health. Nanozyme‐chip systems have emerged as a promising tool for this purpose, enabling the sensitive analysis of nutrients such as vitamins and glucose.

Ascorbic acid exhibits excellent antioxidant properties, which contributes to growth promotion and enhanced disease resistance.^[^ ^273^ ^]^ To enable rapid and portable detection of ascorbic acid in food, Guan et al. designed a paper‐based colorimetric system (Figure 9d).^[^ ^274^ ^]^ In their design, AuNPs nanozyme mimic POD activity to catalyze H_2_O_2_‐mediated oxidation of ABTS to produce a green signal. The reducing properties of ascorbic acid convert the aforementioned green oxidation product ox‐ABTS back to the colorless reduced state ABTS. The resulting colorimetric signal enables visual detection on paper‐based microfluidic chips. By integrating a smartphone‐based quantitative system, this approach completes the testing of beverages and fruits within 30 min. Moreover, the paper‐based microfluidic chip is cost‐effective and well‐suited for large‐scale on‐site screening.

Glucose serves as a crucial energy source for the human body, yet excessive intake can disrupt metabolic balance and lead to chronic diseases such as obesity, diabetes, and cardiovascular disorders.^[^ ^275^, ^276^ ^]^ Consequently, accurate detection of glucose content in food is essential for maintaining dietary quality. Recently, Chen et al. developed a nanozyme‐biochip system by integrating AuNPs as mimetic enzymes, paper‐based microfluidic chip as a portable platform, and a smartphone as analytical terminal.^[^ ^277^ ^]^ The AuNPs are capable of catalyzing the reaction between glucose and H_2_O_2_ oxidase (GOx), which facilitates the decomposition of H_2_O_2_. The generated·OH then oxidize ABTS to produce a distinct green color. This color change was captured and analyzed via the smartphone, enabling intelligent, portable, and low‐cost detection of glucose in fruits and beverages.

In conclusion, nanozyme‐biochip system has the potential to address the need for rapid and on‐site food testing (**Table** **4**). Especially, intelligent detection systems are gaining increased attention owing to their high efficiency and precision. The integration of AI with nanozyme‐biochip systems presents a promising direction for the future development of food safety monitoring. Building on their role in individual and population health, these platforms are increasingly applied to environmental monitoring, reflecting a natural expansion of scope from human well‐being to ecological sustainability.

**Table 4:** Food testing by nanozyme‐biochip systems.

Application type	Analyte	Nanozymes	Nanomaterials	Chip features	Detection range	LOD	Refs.
Foodborne pathogen	*S. typhimurium*	POD	Pt‐AMP	Colorimetry	1×10^2^–1×10^8^ CFU mL^−1^	27 CFU mL^−1^	[251]
	*Salmonella*	POD	MnO_2_ NFs	Colorimetry	44–4.4×10^6^ CFU mL^−1^	44 CFU mL^−1^	[194]
	*Salmonella*	POD	Au@PtPd	Colorimetry	45–4.5×10^6^ CFU mL^−1^	45 CFU mL^−1^	[254]
Mycotoxin	AFB_1_	CAT	PtNPs	Colorimetry	0–60 nM	6.13 nM	[260]
	OTA	POD	Pd@PCN‐222	Electrochemical	0.005–50 ng mL^−1^	1.21 pg mL^−1^	[262]
Pesticide residue	OP	POD	CeGONRs^a)^	Colorimetry	0.012–3.5 mg mL^−1^	3.43 ng mL^−1^	[265]
	CBF	POD	SAzyme	Colorimetry	10–500 ng mL^−1^	8.79 ng mL^−1^	[188]
	GLY	POD	ZnCo‐ZIFs@MIL‐101(Fe)	Colorimetry	0–1 µg mL^−1^	1 ng mL^−1^	[270]
Nutrients	Ascorbic Acid	POD	AuNPs	Colorimetry	0.01–0.125 mmol L^−1^	0.406 µmol L^−1^	[274]
	Glucose	POD	AuNPs	Colorimetry	0.05–5.25 mmol L^−1^	7.615 µmol L^−1^	[277]

^a)^ Ce(III)‐driven self‐assembled strategy to fabricate nanocomposites.

### 5.3 Environmental monitoring

Extending beyond human and food health, nanozyme‐biochip systems are increasingly applied to environmental monitoring, emphasizing their relevance for ecological protection and sustainability. Environmental pollution has become a critical challenge impeding sustainable development and threatening population health.^[^ ^278^ ^]^ While large‐scale instruments exist, they are limited by high cost, slow response, and lack of on‐site capability.^[^ ^279^ ^]^ In contrast, nanozyme‐biochip systems provide intelligent, high‐throughput, and portable detection of pollutants such as heavy metal ions, phenolic compounds, and toxins, driving significant advancements in environmental monitoring.

#### 5.3.1 Heavy metal ions

Heavy metal ions (e.g., Hg^2+^, Pb^2+^, Cu^2+^, Ag^+^, and Cd^2+^) are not readily metabolized when they accumulate in the body through the food chain and may deposit in specific organs, causing chronic toxicity.^[^ ^280^ ^]^ Therefore, accurately measuring the concentrations of heavy metal ions in the environment is of paramount importance for safeguarding human health and the ecosystem. Nanozyme‐biochip systems offer high sensitivity, rapid response, and low cost, making them particularly suitable for on‐site detection of heavy metal ions.

**Figure 10:**
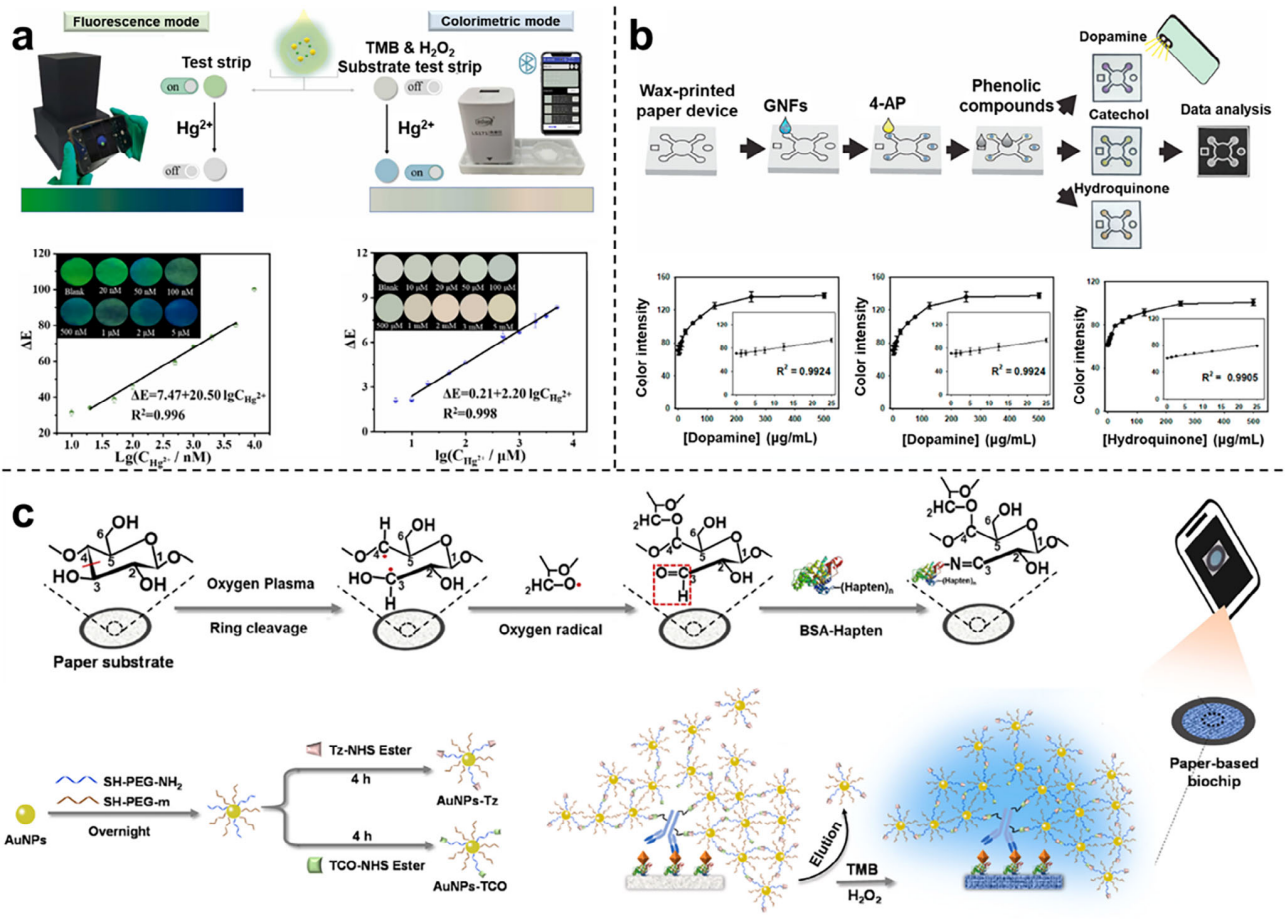
Nanozyme‐biochips for environmental monitoring. a) Schematic diagram of a portable dual‐mode FL and colorimetric sensor for Hg^2+^ detection. Reproduced with permission.^[^ ^285^ ^]^ Copyright 2024 Elsevier. b) Paper‐based microfluidic device for colorimetric detection of phenolic compounds, including dopamine, catechol, and hydroquinone. Reproduced with permission.^[^ ^295^ ^]^ Copyright 2021 Elsevier. c) A signal‐enhanced nanozyme‐biochip system for detecting cyanotoxins in water. Reproduced with permission.^[^ ^298^ ^]^ Copyright 2022 Elsevier.

With the rapid development of industry, Hg^2+^ pollution has become increasingly severe and poses a significant environmental threat.^[^ ^281^, ^282^ ^]^ Wang et al. constructed an electrochemical chip using gold‐modified thiol‐reduced graphene (Au@HS‐rGO) nanozyme, which enabled highly sensitive detection of Hg^2+^.^[^ ^283^ ^]^ However, electrochemical detection methods are susceptible to interference from non‐target substances in samples. In contrast, colorimetric methods offer high specificity and become the commonly employed techniques for detecting Hg^2+^.^[^ ^284^ ^]^ Based on POD‐like gold nanoclusters (Au NCs) nanozyme, Luo et al. developed a paper‐based chip for the colorimetric detection of Hg^2+^.^[^ ^285^ ^]^ However, the Au NCs exhibit relatively low activity compared to natural enzymes. To enhance nanozyme activity, Luo et al. developed a Ce‐aggregated gold nanocluster (Ce‐Au NCs), which generates excellent POD‐like activity, produce strong signal, and enable specific recognition. By integrating with paper‐based biochip, this system achieves portable fluorescence (FL) and colorimetric dual‐mode sensing (**Figure** **10**a). In the presence of Hg^2+^, fluorescence was effectively quenched, and the chips gradually darkened from green to colorless, while colorimetric signal was significantly enhanced accompanied. In practical applications for on‐site detection of agricultural irrigation water, this device connects to a smartphone via Bluetooth and utilizes a colorimeter to collect and analyze experimental results, enabling quantitative on‐site detection of Hg^2+^.

In addition to Hg^2+^, Pb^2+^ is another prevalent heavy metal pollutant in the natural environment, and poses serious risks to human health.^[^ ^286^ ^]^ For accurate detection of Pb^2+^, Li et al. developed a novel fluorescent sensor that integrates a spherical nucleic acid (SNA) probe with the CRISPR‐Cas12a system.^[^ ^287^ ^]^ The SNA probe, which is constructed from gold nanoparticles and DNAzymes, provides specific recognition of Pb^2+^ and exhibits strong nuclease resistance, ensuring reliable performance in complex sample matrices. CRISPR‐Cas12a further enhances detection sensitivity through fluorescent signal amplification. Furthermore, by incorporating a portable photonic crystal chip with a smartphone, this system achieved on‐site monitoring of Pb^2+^ in air particles and soil samples.

Due to the complex composition of pollutants, existing nanozyme‐biochip systems are also evolving toward the simultaneous detection of multiple ions. In a related study, Chen et al. developed a smartphone‐assisted fluorescence and colorimetric approach for detecting Hg^2+^ and Cl^−^ based on the OXD‐like activity of Au‐Hg alloy supported on Au/Cu/ Ti_3_C_2_ nanosheets.^[^ ^288^ ^]^ The presence of Hg^2+^ stimulates the catalytic activity of the nanozyme, generating a fluorescence peak at 570 nm, while Cl^−^ inhibits the catalytic activity and extinguishes the fluorescence signal. This method detects target ions through observable color changes and utilizes smartphone‐based color recognition software for digital image analysis, enabling convenient on‐site monitoring.

#### 5.3.2 Phenolic Compound

Phenolic compounds, such as phenol, chlorophenol, bisphenol A, are primarily released from industrial waste. These compounds exhibit high resistance to degradation and possess the potential to disrupt biological functions, thereby posing risks to human health through digestive, respiratory, and dermal exposure.^[^ ^289^, ^290^ ^]^ Nanozyme‐biochip systems contribute to the simple, accurate, and rapid detection of phenolic compounds in the environment.

Bisphenol A (BPA) is a common industrial chemical frequently utilized in the synthesis of polycarbonate plastics, epoxy resins, and polyester resins. Its pervasive presence in environment poses significant risks to both ecological systems and human health.^[^ ^291^, ^292^ ^]^ Consequently, an urgent need exists to develop cost‐effective and on‐site detection methods. To meet the requirement, Kong et al. developed a portable detection strategy leveraging the intrinsic POD‐like activity of ZnFe_2_O_4_ magnetic nanoparticles (ZnFe_2_O_4_ MNPs) combined with the selective adsorption capability of molecularly imprinted polymer membranes.^[^ ^293^ ^]^ In the presence of BPA, the ZnFe_2_O_4_ MNPs catalyze a chromogenic reaction, producing a color shift with intensity proportional to BPA concentration. This color change is visible to the naked eye and can be quantitatively analyzed using imaging software. By utilizing low‐cost paper‐based components, this colorimetric sensor offers a promising tool for portable BPA monitoring in applications such as environmental assessment and security inspection.

Moreover, to facilitate the efficiency of environmental monitoring, researchers have integrated sensors with smartphones for mobile data transmission.^[^ ^294^ ^]^ For instance, Tran et al. achieved rapid phenolic compound detection by analyzing smartphone‐captured images with ImageJ software.^[^ ^295^ ^]^ In their work, DNA‐copper hybrid nanoflowers (GNFs) were fabricated through a straightforward self‐assembly of guanine‐rich single‐stranded DNA and copper phosphate. As shown in Figure 10b, GNFs catalyze the reaction between phenolic compounds and 4‐aminoantipyrine to produce colored products on chip. Meanwhile, GNFs exhibit high stability and diversity oxidation capability toward diverse phenolic compounds, including dopamine, catechol, and hydroquinone. This method requires only 50 µL of sample and completes the entire process within 20 min, showing significant potential for applications in biosensors and bioremediation.

#### 5.3.3 Toxin

Environmental toxins, which are mainly caused by cyanobacteria in freshwater systems, such as microcystins (MCs) and nodularins (NODs), pose severe threats to human health and ecosystems.^[^ ^296^ ^]^ Nanozyme‐biochip systems show considerable promise for potential application in detecting these toxins.

To detect microcystin‐RR (MC‐RR) in water samples, Han et al. developed a novel microfluidic paper‐based colorimetric sensor using molecular imprinting technology.^[^ ^297^ ^]^ Based on Fenton reaction and colorimetric reaction between TMB and H_2_O_2_, the system enables smartphone‐assisted image and software‐based quantification of MC‐RR, providing a precise and intelligent approach for on‐site detection of MC‐RR.

In addition, cyanotoxins exhibit considerable diversity. To detect broad ranges of MCs and NODs, Liu et al. developed a nanozyme‐enhanced immunochip system integrated with smartphone readout (Figure 10c).^[^ ^298^ ^]^ This approach utilizes broad‐spectrum monoclonal antibodies to simultaneously recognize multiple MC and NOD variants. The integration of biorthogonal click reaction with nanozyme‐catalyzed signal amplification technology enables the sensitive detection of 13 MC or NOD variants within 60 min, with detection limits below 0.7 µg L^−1^ for all variants. This method offers an affordable, robust solution for on‐site monitoring and large‐scale screening of cyanotoxins in water.

In summary, with the advantages of low cost, portability, and strong anti‐interference capacity, nanozyme‐biochip systems exhibit exceptional performance in the detection of heavy metal ions, phenolic compounds, and toxins (**Table** **5**). They represent a powerful candidate for meeting the critical demands of on‐site environmental monitoring and are poised to play a pivotal role in supporting ecological conservation and environmental sustainability. Taken together, the progression from disease diagnosis to food safety and finally to environmental monitoring illustrates a coherent translational pathway, underscoring the broad societal relevance of nanozyme‐biochip systems.

**Table 5:** Environmental monitoring by nanozyme‐biochip systems.

Application type	Analyte	Nanozymes	Nanomaterials	Chip features	Detection range	LOD	Refs.
Heavy metal ion	Hg^2+^	POD	Ce‐Au NCs	Fluorescence	20 nM–5 µM	20 nM	[285]
				Colorimetry	10 µM–5 mM	10 µM	
	Pb^2+^	Nuclease	DNAzyme	Fluorescence	0.1 pM–1 µM	86 fM	[287]
	Hg^2+^	OXD	Au/Cu/Ti_3_C_2_ NSs	Colorimetry	8–200 nM	0.8 nM	[288]
	Cl^−^				3–350 µM	3 µM	
Phenolic compounds	Bisphenol A	POD	ZnFe_2_O_4_ MNPs	Colorimetry	10–1000 nM	6.18 nM	[293]
	Dopamine	OXD	DNA‐copper hybrid	Colorimetry	5–500 µg mL^−1^	4.5 µg mL^−1^	[295]
	Catechol					3 µg mL^−1^	
	Hydroquinone					4.5 µg mL^−1^	
Toxin	Microcystins RR	POD	ZnFe_2_O_4_@SMIPs µPADs	Colorimetry	1–100 µg L^−1^	0.44 µg L^−1^	[297]
	MCs/NODs	POD	AuNPs‐TCO	Colorimetry	0.52–28.88µg L^−1^	0.7 µg L^−1^	[298]

## 6 Discussion of Nanozyme‐Biochip Systems

Nanozyme‐biochip systems demonstrate remarkable advantages when compared with traditional enzyme‐based biosensors (**Table** **6**). Compared with natural enzymes, nanozymes effectively address critical limitations, including poor enzymatic stability, high production costs, and stringent storage requirements. Through rational integration with biochip platforms, nanozyme systems achieve unique multiplexing capabilities through the coupling of multiple detection modalities. Furthermore, the incorporation of AI enables advanced data processing and analysis that substantially surpasses the capabilities of conventional readout systems.

**Table 6:** Performance comparison of nanozyme‐biochip systems with traditional enzyme‐based biosensors and commercial POCT devices.

Parameter	Traditional enzyme‐based biosensor	Commercial POCT device (e.g., glucose strips/lateral‐flow)	Nanozyme‐biochip system
**Detection limit**	≈nM‐pM range High sensitivity	≈µM‐nM range Moderate sensitivity	≈pM‐fM range Ultra‐high sensitivity
**Response time**	30–60 min Complex procedures	5–15 min Rapid operation	1–10 min Rapid operation
**Stability**	Limited enzyme stability Cold chain required	Excellent stability Room temperature storage	Good nanozyme stability Improved thermal resistance
**Cost**	High enzyme cost Expensive reagents	Low manufacturing cost Economical production (≈ USD 2–5/test)	Moderate nanomaterial cost Scalable potential (reusable chip < 0.5 USD /test)
**Advantages**	High specificity Well‐established protocols Clinical validation	User‐friendly Mass production Regulatory approved Portable design	Tunable activity Multi‐enzyme mimicry Miniaturization capability Customizable platforms
**Limitations**	Enzyme instability High cost Specialized storage Limited shelf life	Limited sensitivity Single‐use only Fixed parameters Less customizable	Batch variability Standardization needed Early development stage Regulatory hurdles
**References**	[299, 300]	[301, 302]	[254, 303]

Despite the substantial advances summarized above, nanozyme‐biochip systems continue to face significant challenges in the transition from laboratory to commercial deployment when compared to established POCT devices. These challenges arise from multiple dimensions, including material properties, system integration, signal analysis, and scalable fabrication.

### 6.1 Fundamental Limitations in Material Properties

The functional reliability of nanozymes constitutes a fundamental challenge for commercial applications. While demonstrating excellent catalytic activity in laboratory settings, maintaining this performance under real‐world storage and operational conditions proves difficult due to potential aggregation. Furthermore, achieving batch‐to‐batch consistency in nanozyme synthesis remains problematic, as minor variations in size, shape, and surface chemistry can significantly impact catalytic efficiency and detection reproducibility across different production lots. To address these issues, advanced surface engineering and stabilization strategies are being developed to enhance nanozyme durability, while rigorous quality control protocols are implemented during manufacturing to ensure consistency.

### 6.2 Technical Challenges in System Integration

Substantial technical barriers exist in the effective integration of nanozymes with biochip platforms. Achieving uniform and stable immobilization of nanozymes onto specific regions of microfluidic devices while preserving their catalytic activity remains challenging, particularly when scaling up manufacturing processes. Furthermore, operational reliability is often compromised by practical issues, including microfluidic channel clogging due to nanozyme aggregation or non‐specific adsorption of sample components. Optimizing surface functionalization efficiency and packaging design is therefore crucial for achieving reproducible, certification‐ready prototypes.

### 6.3 Limitations in AI‐Driven Signal Analysis

The implementation of AI for signal processing introduces both opportunities and challenges in commercial translation. Developing robust machine learning algorithms requires extensive training datasets, which can be time‐consuming and resource‐intensive to acquire. Moreover, integrating these computational models into portable devices necessitates balancing processing power with energy consumption. Potential solutions include building open‐access databases and implementing edge‐computing optimized algorithms to maintain accuracy while minimizing computational demands.

### 6.4 Barriers to Scalable Fabrication

Transitioning from laboratory prototypes to mass‐produced commercial devices involves substantial manufacturing challenges. Maintaining consistent performance across different production batches demands careful selection of manufacturing techniques. Emerging micro‐ and nano‐fabrication techniques such as inkjet or aerosol‐jet printing, soft lithography, and laser‐induced 3D patterning provide feasible routes for large‐area integration of nanozymes with biochip. Additionally, a well‐defined approval pathway and standardized evaluation criteria could effectively accelerate technology maturation and market entry.

## 7 Conclusion and Outlooks

The efforts to integrate nanozymes, biochips, and AI have given rise to a novel interdisciplinary paradigm for intelligent biosensing. By combining the high stability and low cost of nanozymes with the miniaturization and integration capabilities of biochips, this system effectively overcomes key limitations of traditional biosensing platforms, including portability, real‐time responsiveness, multiplexed capability, and facile operation. The incorporation of AI further transforms these systems by enhancing post‐signal acquisition processing and accurate analytical functions. These capabilities have substantially expanded the applicability of nanozyme‐biochips in contexts requiring rapid response, high‐throughput analysis, and minimal user intervention, such as clinical diagnostics, food safety, and environmental monitoring.

Looking forward, the integration of nanozyme‐biochip systems with AI is expected to unlock excellent capabilities in biosensing. For example, the achievement of sub‐picomolar detection limits through synergistic advances in signal amplification strategies and interfacial engineering, enabling identification of trace‐level analytes in complex samples. The implementation of on‐chip AI processors with lightweight neural networks and ultra‐low power consumption will facilitate real‐time, intelligent data interpretation for on‐site analysis. Furthermore, roll‐to‐roll manufacturing is projected to reduce production costs, while multi‐analyte platforms will evolve to simultaneously quantify multiple targets on a single chip. These developments, coupled with streamlined regulatory pathways, are expected to accelerate the commercialization of nanozyme‐biochip systems into viable sensing devices.

## Conflict of Interest

The authors declare no conflict of interest.

## Author Contributions

D.C., W.Z., Z.Z., and S.Y. contributed equally to this work. Y.J, Z.D. and L.C. led the whole review. D.C. W.Z., Z.Z., S.Y., X.H, H.W, X.X, W.M. organized and wrote the manuscript.
